# High-Resolution Linkage Map With Allele Dosage Allows the Identification of Regions Governing Complex Traits and Apospory in Guinea Grass (*Megathyrsus maximus*)

**DOI:** 10.3389/fpls.2020.00015

**Published:** 2020-02-26

**Authors:** Thamiris G. Deo, Rebecca C. U. Ferreira, Letícia A. C. Lara, Aline C. L. Moraes, Alessandro Alves-Pereira, Fernanda A. de Oliveira, Antonio A. F. Garcia, Mateus F. Santos, Liana Jank, Anete P. de Souza

**Affiliations:** ^1^ Center for Molecular Biology and Genetic Engineering, University of Campinas, Campinas, Brazil; ^2^ Genetics Department, Escola Superior de Agricultura “Luiz de Queiroz,” University of São Paulo, Piracicaba, Brazil; ^3^ Plant Biology Department, Biology Institute, University of Campinas, Campinas, Brazil; ^4^ Embrapa Beef Cattle, Brazilian Agricultural Research Corporation, Campo Grande, Brazil

**Keywords:** apospory, double reduction, forage, polyploidy, quantitative trait locus, single nucleotide polymorphism, trait correlations

## Abstract

Forage grasses are mainly used in animal feed to fatten cattle and dairy herds, and guinea grass (*Megathyrsus maximus*) is considered one of the most productive of the tropical forage crops that reproduce by seeds. Due to the recent process of domestication, this species has several genomic complexities, such as autotetraploidy and aposporous apomixis. Consequently, approaches that relate phenotypic and genotypic data are incipient. In this context, we built a linkage map with allele dosage and generated novel information of the genetic architecture of traits that are important for the breeding of *M. maximus*. From a full-sib progeny, a linkage map containing 858 single nucleotide polymorphism (SNP) markers with allele dosage information expected for an autotetraploid was obtained. The high genetic variability of the progeny allowed us to map 10 quantitative trait loci (QTLs) related to agronomic traits, such as regrowth capacity and total dry matter, and 36 QTLs related to nutritional quality, which were distributed among all homology groups (HGs). Various overlapping regions associated with the quantitative traits suggested QTL hotspots. In addition, we were able to map one locus that controls apospory (apo-locus) in HG II. A total of 55 different gene families involved in cellular metabolism and plant growth were identified from markers adjacent to the QTLs and APOSPORY locus using the *Panicum virgatum* genome as a reference in comparisons with the genomes of *Arabidopsis thaliana* and *Oryza sativa*. Our results provide a better understanding of the genetic basis of reproduction by apomixis and traits important for breeding programs that considerably influence animal productivity as well as the quality of meat and milk.

## Introduction

Forage grasses play a fundamental role in the global beef production chain. Brazil is the country with the greatest emphasis on this sector, being the main exporter of beef and having the largest commercial herd of beef cattle in the world, with approximately 215 million heads distributed in 162 million hectares of pasture (ABIEC, 2019). The main factor that led to this scenario was the beginning of tropical forage breeding in the 1980s in Brazil, which although recent, permitted the country to become the world's largest exporter of tropical forage seeds (ITC, 2018).

The African forage grass species *Megathyrsus maximus* (Jacq.) B. K. Simon & S. W. L. Jacobs (syn. *Panicum maximum* Jacq.), also known as guinea grass, is one of the most productive forage grasses reproduced by seeds in the Brazilian market and is also grown in other Latin American countries (Jank et al., 2011). It has been used mainly in intensive systems with high-fertility soils (Valle et al., 2009). Moreover, this forage has a high biomass potential and is promising as a biofuel feedstock (Odorico et al., 2018). The polyploidy and domestication process of this forage grass ensure high genetic variability to be explored (Jank et al., 2011); however, a lack of knowledge of the biology and genetics of the species, including its autotetraploidy and facultative apomictic mode of reproduction (Warmke, 1954), may make breeding more difficult and thus stimulate a need to invest in genetic studies.

The polysomic inheritance in autopolyploids makes genetic research difficult because several types of segregation may be involved (Field et al., 2017) and complexity increases as ploidy increases. Thus, in a segregating population, genetic complexity may influence the segregation and the frequencies of expected genotypes (Field et al., 2017), even causing distortions such as double reduction (DR), which is a type of meiosis in which the sister chromatids are duplicated, forming an unexpected combination of gametes. For example, an autotetraploid genotype with the “abcd” alleles at a locus that can form six expected combinations of gametes, namely, “ab”, “ac”, “ad”, “bc”, “bd”, and “cd”, but a homozygous gamete can be generated, e.g., “aa”, “bb”, “cc”, or “dd” (Haldane, 1930; Mather, 1935; Haynes and Douches, 1993). How to accommodate DR and its implications in a breeding program as well as the use of these marker loci in linkage mapping has been discussed for a long time (Butruille and Boiteux, 2000; Luo et al., 2000; Xu et al., 2013; Layman and Busch, 2018; Bourke et al., 2019) for some economically important species, such as potato (Bradshaw, 2007; Bourke et al., 2015) and alfalfa (Julier et al., 2003), but has not yet been reported in guinea grass.

Linkage maps have been used as the primary source of genetic information for nonmodel species that do not have their genome sequenced, such as *M. maximus*. The construction of dense linkage maps allows the identification of the structure and evolution of the genome by mapping traits with polygenic and monogenic inheritance and may even contribute to the assembly of the genome of a species (Doerge, 2002; Flint-Garcia et al., 2003; Luo et al., 2004). The majority of linkage maps available for autotetraploids are based on single-dose segregating markers for a parent (Aaaa x aaaa) or a single dose for both parents (Aaaa x Aaaa). Despite the use of complex statistical methods to obtain integrated maps that combine information from both marker patterns, these maps cover only part of the genome because higher-dose markers (AAaa, AAAa, and AAAA) are not included. This limitation results in a considerable loss of genetic information. To overcome this limitation, a new approach allows the assignment of allele dosage information for single nucleotide polymorphism (SNP) markers through exact allele sequencing depth, which generates linkage maps from markers in multiple doses with higher quality, more information and greater applicability, including more efficient detection of loci related to traits of economic importance (Serang et al., 2012; Hackett et al., 2014; Pereira et al., 2018; Mollinari and Garcia, 2019).

Mapping of the aposporous apomixis region is extremely important for the genetic breeding of *M. maximus* and other forage grasses, such as *Urochloa* spp., *Paspalum* spp., and *Cenchrus ciliaris*. These species undergo asexual propagation by seeds (Nogler, 1984), which allows the fixation of hybrid vigor in apomictic individuals and their use in the creation of uniform pastures (Jank et al., 2011). Experimental field data showed that aposporous apomixis in tropical forage grasses follows 1:1 Mendelian segregation, indicating monogenic inheritance (Savidan, 1981; Valle et al., 1994; Chen et al., 2000; Savidan, 2000), although a recent study suggests that this reproductive mode should be treated as a quantitative trait (Marcón et al., 2019), and provide evidence of the uncoupling of apomixis in neo-apomictic species, such as guinea grass (Kaushal et al., 2008; Kaushal et al., 2019). Molecularly, the apospory-specific genomic region (ASGR), which is responsible for apospory, is highly conserved among apomictic species (Gualtieri et al., 2006). The influence of some factors, such as epigenetics (Kumar, 2017), the presence of retrotransposons (Akiyama et al., 2011), and gene duplication (Conner et al., 2008), in this region has been reported for other species. Due to the laborious and time-consuming methods required to phenotype apomixis, several studies on forage grasses have searched for markers intrinsically linked to the chromosomal region for this trait (Pessino et al., 1998; Ebina et al., 2005; Bluma-Marques et al., 2014; Thaikua et al., 2016; Vigna et al., 2016; Worthington et al., 2016; Worthington et al., 2019); however, a 100% efficient marker for use in *M. maximus* breeding programs has not yet been identified.

In guinea grass there are no mapping studies of loci related to complex traits, such as those involved in forage yield and nutritional quality. Forage yield results from the continuous emission of leaves and tillers, ensuring the restoration of the leaf area after grazing in perennial pastures. Additionally, the nutritional value of a forage is directly related to animal performance and is measured by the crude protein, *in vitro* digestibility, neutral and acid detergent fiber, and lignin percentages (Jank et al., 2011). Thus, the mapping of these and other important quantitative trait loci (QTLs) may provide information about the genetic architecture of traits and assist in new strategies for breeding programs of *M. maximus*.

In this context, given the importance of new genomic studies in guinea grass for both biological knowledge and support for breeding programs, our goals were to (i) construct an integrated consensus linkage map from a full-sib progeny of *M. maximus* using allele dosage information, (ii) detect QTLs related to important agronomic and nutritional traits in this progeny, (iii) map the apo-locus, and (iv) search for similarity in regions of the markers adjacent to the QTLs and APOSPORY locus in *Arabidopsis thaliana, Panicum virgatum* and *Oryza sativa*.

## Material and Methods

### Plant Material

A full-sib progeny of 136 F_1_ hybrids was obtained from a cross between a facultative apomictic genotype of *M. maximus* cv. Mombaça and an obligate sexual genotype, S10. For the crosses, 100 m^2^ of cv. Mombaça was sown in lines with a space of 1 m between the lines. One plant of S10 was planted in each 5 m x 5 m grid. The sexual plants were monitored to ensure the synchronization of flowering with the cv. Mombaça, and the inflorescences that flowered before cv. Mombaça were cut. According to Savidan (1982), 25 m^2^ of the apomictic parent is sufficient to pollinate one sexual plant and to impede contamination from neighboring plants. The S10 plants are wind-pollinated, and the S10 seeds were harvested as they matured.

The sexual parent was derived from sexual x apomictic crosses of an original diploid sexual plant that was duplicated with colchicine (Savidan and Pernès, 1982); thus, both parents were autotetraploid (2n = 4x = 32) (Savidan et al., 1989). In addition to the reproductive mode, the parents have contrasting agronomic and nutritional quality traits (Braz et al., 2017). S10 is a medium sized plant (1.4 m tall) with medium width leaves (2.4 cm wide). Both its leaves and stems are glabrous, and its inflorescences consist of a panicle with short primary ramifications and long secondary ramifications throughout. Its spikelets are glabrous and purplish. The cv. Mombaça is a tall plant (1.7 m tall) with wide leaves (3 cm). Its leaves have small amount of hairs, and its stems are glabrous. Its inflorescences comprise a panicle with short primary ramifications and long secondary ramifications only on the inferior ramifications, and its spikelets are glabrous and light purple.

DNA extraction followed the protocol described by Doyle and Doyle (1987), with modifications. DNA samples were visualized on 2% agarose gels to check their quality and integrity, and their concentrations were estimated using a Qubit 3.0 fluorometer (Thermo Scientific, Wilmington, USA).

To retain only true full-sibs, all possible hybrids were previously genotyped with microsatellite markers. This analysis revealed 24 false hybrids, which were excluded at the construction stage of the GBS library.

### Experimental Design

A field experiment following an augmented block design (ABD) with 160 regular treatments (full-sib progeny) and two checks (the parents ‘Mombaça’ and S10) distributed in eight blocks with two whole replicates was performed at Embrapa Beef Cattle (Brazilian Agricultural Research Corporation), in Campo Grande city, Mato Grosso do Sul state, Brazil (20°27ʹS, 54°37ʹW, 530 m). Each block consisted of a total of 22 plots (20 individuals and two checks).

Each plant was evaluated for agronomic and nutritional quality traits, totaling 22 traits: i) agronomic traits: green matter (GM—g/plant), total dry matter (TDM—g/plant), leaf dry matter (LDM—g/plant), stem dry matter (SDM—g/plant), regrowth capacity (RC), and percentage of leaf blade (PLB—%) and ii) nutritional quality traits for the leaf and stem: organic matter (OM_L and OM_S, respectively—%), crude protein (CP_L and CP_S—%), *in vitro* digestibility of organic matter (IVD_L and IVD_S—%), neutral detergent fiber (NDF_L and NDF_S—%), acid detergent fiber (ADF_L and ADF_S—%), cellulose (CEL_L and CEL_S—%), silica (SIL_L and SIL_S—%), and permanganate lignin (PL_L and PL_S—%). The agronomic traits were evaluated for six harvests (three harvests in 2013 and three harvests in 2014), but RC was evaluated for only three harvests (one harvest in 2013 and two harvests in 2014). The nutritional quality traits were evaluated for only one harvest in 2014.

### Statistical Analysis of Phenotypic Data

Descriptive analyses were performed, and the Box-Cox transformation (Box and Cox, 1964) was applied to correct for nonnormality of the residuals. For traits with multiple harvests (agronomic traits), we fitted the following longitudinal linear mixed model:

$$y_{ijkl}=\text{μ}+h_{l}+r_{k(l)}+b_{j(l)}+rb_{kj(l)}+t_{i(l)}+\epsilon_{ijkl}$$

where *y_ijkl_* was the phenotypic value of the *i^th^* treatment in the *j^th^* block and *k^th^* replicate at the *l^th^* harvest; µ was the fixed overall mean; *h_l_* was the fixed effect of the *l^th^* harvest (*l* = 1, ..., *L*, with *L* = 3 for RC and *L* = 6 for the other traits); *r_k(l)_* was the fixed effect of the *k^th^* replicate (*k* = 1, ..., *K*, with *K* = 2) at harvest *l*; *b_j(l)_* was the random effect of the *j^th^* block (*j* = 1, ..., *J*, with *J* = 8) at harvest *l*, with *b_j(l)_* ~ *N*(0,$\sigma_{b}^{2}$); *rb_kj(l)_* was the random interaction effect of replication *k* and block *j* at harvest *l*, with *rb_kj(l)_* ~ *N*(0,$\sigma_{rb}^{2}$); *t_i(l)_* was the effect of the *i^th^* treatment (*i* = 1, ..., *I*, with *I* = 162) at harvest *l*; and *ε_ijkl_* was the random environmental error. The treatment effects (*t_i(l)_*) were separated into two groups: *g_i(l)_* was the random effect of the *i^th^* individual genotype (*i* = 1, ..., *I_g_*, with *I_g_* = 160) at harvest *l*, and *c_i(l)_* was the fixed effect of the *i^th^* check (*i* = 1, ..., *I_c_*, with *I_c_* = 2) at harvest *l*. For genotype effects, the vector ***g*** = (*g_11_*, ..., *g_IgL_*)` was assumed to follow a multivariate normal distribution with a mean of zero and genetic variance-covariance (VCOV) matrix ***G*** = ***G_L_*** Ⓧ ***I_Ig_***, i.e., ***g*** ~ *N*(**0**, ***G***). For residual effects, the vector ***ε*** = (*ε*_1111_, ..., *ε_IJKL_*)` followed a multivariate normal distribution with a mean of zero and residual VCOV matrix ***R*** = ***R_L_*** Ⓧ ***I_I_***.***_J_***.***_K_***, i.e., ***ε*** ~ *MVN*(**0**, ***R***).

The VCOV matrices ***G_L_*** and ***R_L_*** were analyzed considering seven different structures: identity (ID), diagonal (DIAG), compound symmetry (CS), heterogeneous compound symmetry (CS_Het_), first-order autoregressive (AR1), heterogeneous first-order autoregressive (AR1_Het_), first-order factor analytic (FA1), and unstructured (US). First, the genetic VCOV matrix (***G_L_***) was analyzed considering the ID for the residual matrix (***R_L_***), and posteriorly, the residual matrix (***R_L_***) was analyzed considering the selected VCOV matrix for genetic effects. Model selection was performed based on the Akaike information criterion (AIC) (Akaike, 1974) and Schwarz information criterion (SIC) (Schwarz, 1978).

For the nutritional quality traits, we fitted the following linear mixed model:

$$y_{ijk}=\mu +r_{k}+b_{j}+rb_{kj}+t_{i}+\epsilon_{ijk}$$

where *y_ijk_* was the phenotypic value of the *i^th^* treatment in the *j^th^* block and *k^th^* replication; µ, *r_k_*, *b_j_*, *rb_kj_*, *t_i_*, and *ε_ijk_* were as described above but not nested within harvest and with *ε_ijk_* ~ *N*(0,$\sigma_{\epsilon }^{2}$). The treatment effects (*t_i_*) were separated into two groups: *g_i_* as a random effect, *g* ~ *N*(0,$\sigma_{g}^{2}$), and *c_i_* as a fixed effect. All analyses were performed with the R package ASReml-R (Butler et al., 2009).

The heritability of each trait was calculated using the same model as previously mentioned but considering the ***G_L_*** and ***R_L_*** matrices as the ID. The equation was

$${\hat{H}}^{2}=\frac{\sigma_{g}^{2}}{\sigma_{P}^{2}}$$

where $\sigma_{g}^{2}$ is the genetic variance and $\sigma_{P}^{2}$ is the phenotypic variance. The network analysis was carried out using the R package ‘qgraph' (Epskamp et al., 2012).

### Identification of the Reproductive Mode

The aposporic or sexual reproductive mode was determined for 106 hybrids of the progeny (**Supplementary Table 1**). From the flowers collected during anthesis, we performed an analysis of 30 ovules per hybrid using the clarified ovary method described by Young et al. (1979). Nomarski differential interference contrast microscopy was used to view the ovaries. A chi-square test was performed to verify the Mendelian segregation of this trait according to the expected model of monogenic inheritance in the base package of R (version 3.5.0) (R Core Team, 2018).

### GBS Library Preparation and Sequencing

From the extracted DNA, genotyping-by-sequencing (GBS) libraries were built according to Poland et al. (2012), containing 12 replicates for each parent. A total of 200 ng of genomic DNA per sample was digested with a combination of a rare-cutting enzyme (*Pst*I) and a frequently cutting enzyme (*Msp*I). DNA fragments were ligated to the common and barcode adapters, and the libraries were sequenced as 150-bp single-end reads using the High Output v2 Kit (Illumina, San Diego, CA, USA) for the NextSeq 500 platform (Illumina, San Diego, CA, USA).

### SNP Calling and Allele Dosage Analysis

First, raw data were checked for quality using NGS QC Toolkit (Patel and Jain, 2012). SNP calling analysis was performed using the TASSEL-GBS v.4 pipeline (Glaubitz et al., 2014) modified for polyploids (Pereira et al., 2018) that use exact read depths. The default parameters were changed as follows: the minimum number of times a GBS tag must be present was changed to 5, and the minimum count of reads for a GBS tag was changed to 2. This pipeline requires a genome as a reference for SNP calling, but no genome sequence of *M. maximus* is available. To overcome this limitation, the switchgrass genome (*P. virgatum* v1.0, produced by the US Department of Energy Joint Genome Institute) available in the Phytozome database (http://phytozome.jgi.doe.gov/) (Goodstein et al., 2012) was chosen because this species is phylogenetically closely related to *M. maximus* (Burke et al., 2016). GBS tags were aligned to the reference genome with Bowtie2 2.3.1 (Langmead et al., 2009) using the following settings: very-sensitive-local, a limit of 20 dynamic programming problems (D) and a maximum of 4 times to align a read (R). Subsequently, only tags that aligned exactly one time were processed. Then, SNP calling was performed under the conditions that the minor allele frequency was greater than 0.05 and the minor allele count was greater than 1,000. Mismatches of duplicated SNPs greater than 0.2 were not merged. Then, in R software (version 3.5.0) (R Core Team, 2018), we selected only the SNPs with a minimum average allele depth equal to or greater than 60 reads. The updog package (Gerard et al., 2018) was used to estimate the allele dosage of these markers, with a fixed ploidy parameter of 4 and the flexdog function considering the F_1_ population model. SNPs with less than 0.15 of the posterior proportion of individuals incorrectly genotyped were selected. GBS sequences of each individual were deposited in the NCBI database under number PRJNA563938.

### Quality Filtering of SNPs

We removed markers with more than 25% missing data and monomorphic markers manually in R software (version 3.5.0) (R Core Team, 2018). Subsequently, we followed Bourke et al. (2018a) to ensure the retention of reliable markers. We first verified the shifted markers for the polysomic inheritance model from which SNP markers that did not correspond to an expected segregation type were removed. A threshold of 5% was used for missing values per marker and per individual. Duplicated markers, which provided no extra information about a locus, were also removed in this step. Finally, a principal component analysis (PCA) was performed to identify individuals who deviated from the progeny as well as possible clones.

### Linkage Map Construction

A linkage map was constructed using TetraploidSNPMap version 3.0 (Hackett et al., 2017), which allows the use of SNP markers with allele dosage data for autotetraploid species. SNP markers were checked with a chi-square test for goodness of fit, and only markers with a simplex configuration value greater than 0.001 and a segregation value greater than 0.01 for higher dosage were selected for mapping. Some unselected markers were classified as having segregation distortion (SD), being incompatible with the parental dosages (NP) and having DR. To order the selected markers, two-point analysis and multidimensional scaling analysis (MDS) were used to calculate recombination fractions and logarithm of odds (LOD) scores. Outlier markers were removed in this step. Some phases of the linked SNPs were inferred by TetraploidSNPMap software, and other phases were determined manually. The integrated consensus map represented by homology groups (HGs) was plotted using MapChart 2.32 (Voorrips, 2002), in which SNP configurations were identified with different colors.

### Monogenic and Polygenic Trait Analysis

QTL mapping of six agronomic traits and sixteen nutritional quality traits was performed with TetraploidSNPMap, applying an interval mapping model (Hackett et al., 2014; Hackett et al., 2017). Analyses were conducted for each HG separately using three data files: phenotypic trait data, genotypic data and map data with phase information. The phenotypic data for the reproductive mode, i.e., aposporic or sexual, were considered qualitative due to the evaluation method applied; i.e., apomictic individuals were coded as one, and sexual individuals, as zero. The other phenotypic traits were analyzed as quantitative. QTL positions and significance were evaluated with a 1,000 permutation test. A QTL was declared significant if its LOD score was above the 90% threshold. Simple models were tested for each significant QTL to verify the best QTL model. The lowest SIC (Schwarz, 1978) was the criterion used to define the best model. Using TetraploidSNPMap software, if two or more significant QTLs were identified on the same chromosome, only the one with the greatest effect was considered.

### Search for Similarity in Aposporic and QTL Regions

We performed a search for similarity of candidate genes located close to the detected QTL/apospory locus regions. Using the switchgrass genome as a reference and based on chromosomal locations of the markers adjacent to the detected QTLs and apospory locus, we aligned the sequences found in 100-kb regions with Basic Local Alignment Search Tool (BLAST) (e-value cutoff of 1e-0.5) against the *A. thaliana* and *O. sativa* genomes through the JBrowse tool in Phytozome (http://phytozome.jgi.doe.gov/) (Goodstein et al., 2012).

## Results

### Phenotypic Data

Different VCOV matrices were selected for the agronomic and nutritional traits (**Supplementary Tables 2** and **3**). When the AIC and SIC were not in agreement, we selected the matrices based on the largest difference between the models. For example, considering the GM trait and the ***G_L_*** matrix, US was selected based on the AIC, and CS was selected based on the SIC (US had 567.32 for the AIC and 702.21 for the SIC, and CS had 581.66 for the AIC and 598.53 for the SIC). The differences between these two selected models were 14.34 for the AIC (581.66-567.32) and 103.68 for the SIC (702.21-598.53). As the SIC produced the largest difference, this criterion was used, and the CS matrix was selected for the ***G_L_*** matrix of the GM trait. The heritabilities ranged from 0.19 (PLB) to 0.64 (GM) for the agronomic traits and from 0.06 (SIL_S) to 0.31 (OM_L and CEL_S) for the nutritional quality traits (**Table 1**). Box-Cox transformation was performed for agronomic traits (GM, TDM, LDM, SDM, RC, PLB) and nutritional traits of the leaves and stems (OM_L, OM_S, CP_S, IVD_L, IVD_S, NDF_S, ADF_L, CEL_L, SIL_L, SIL_S, PL_L, and PL_S).

**Table 1:** Broad-sense heritability obtained for the agronomic and nutritional traits for the F1 mapping population of guinea grass (*Megathyrsus maximus*) evaluated in this study.

Traits		H²
**Agronomic**	Green matter (GM)	0.64
	Total dry matter (TDM)	0.57
	Leaf dry matter (LDM)	0.58
	Stem dry matter (SDM)	0.35
	Percentage of leaf blade (PLB)	0.19
	Regrowth capacity (RC)	0.36
**Nutritional**	Leaf organic matter (OM_L)	0.31
	Stem organic matter (OM_S)	0.15
	Leaf crude protein (CP_L)	0.13
	Stem crude protein (CP_S)	0.28
	Leaf neutral detergent fiber (NDF_L)	0.14
	Stem neutral detergent fiber (NDF_S)	0.14
	Leaf acid detergent fiber (ADF_L)	0.25
	Stem acid detergent fiber (ADF_S)	0.30
	Leaf in vitro digestibility of organic matter (IVD_L)	0.18
	Stem in vitro digestibility of organic matter (IVD_S)	0.26
	Leaf cellulose (CEL_L)	0.26
	Stem cellulose (CEL_S)	0.31
	Leaf permanganate lignin (PL_L)	0.18
	Stem permanganate lignin (PL_S)	0.18
	Leaf silica (SIL_L)	0.17
	Stem silica (SIL_S)	0.06

**Figure 1:**
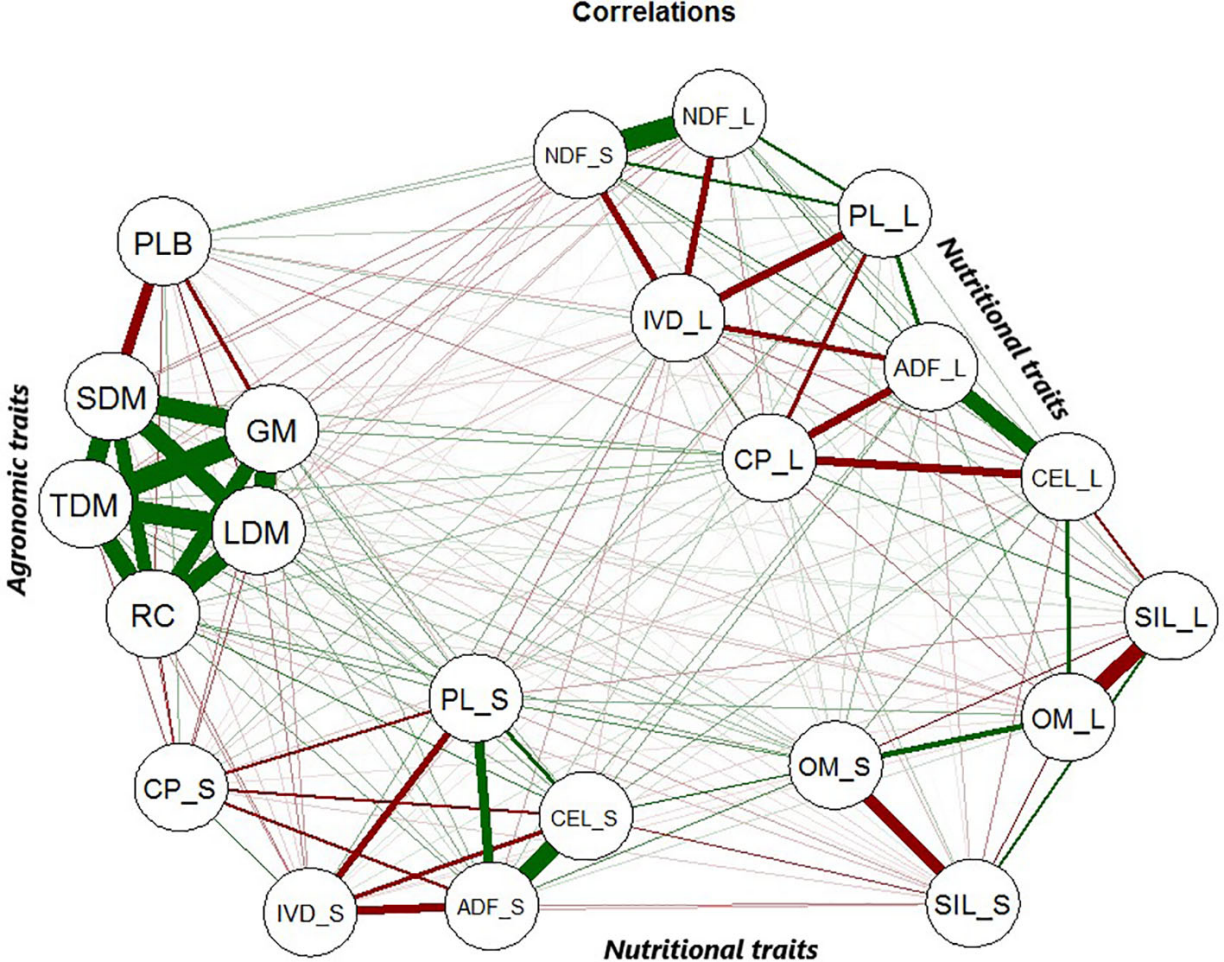
Correlations among all phenotypic traits from the guinea grass mapping population. The green lines correspond to positive correlations between the traits, and the red lines correspond to negative correlations between the traits. The agronomic traits were green matter (GM), total dry matter (TDM), leaf dry matter (LDM), stem dry matter (SDM), percentage of leaf blade (PLB), and regrowth capacity (RC). The leaf and stem nutritional quality traits were the following: organic matter (OM_L and OM_S), crude protein (CP_L and CP_S), neutral detergent fiber (NDF_L and NDF_S), acid detergent fiber (ADF_L and ADF_S), in vitro digestibility of organic matter (IVD_L and IVD_S), cellulose (CEL_L and CEL_S), permanganate lignin (PL_L and PL_S), and silica (SIL_L and SIL_S).

The correlations between the agronomic and nutritional traits are presented in **Figure 1**. Significant and positive correlations were observed among GM, TDM, SDM, LDM, and RC.

PLB presented a negative correlation with other agronomic traits and a positive correlation with NDF, but these correlations were weaker than those found for the other pairs of traits. Complex correlations between nutritional traits were obtained. In particular, stem-related traits were most tightly correlated with leaf-related traits, and NDF_L and NDF_S were closely correlated. The ADF, CEL and PL traits exhibited a significant and positive correlation. The same pattern was observed for the leaf and stem nutritional traits: CP and IVD exhibited a strong negative correlation with PL, ADF, CEL and NDF.

### Genotyping and Linkage Analysis

**Figure 2:**
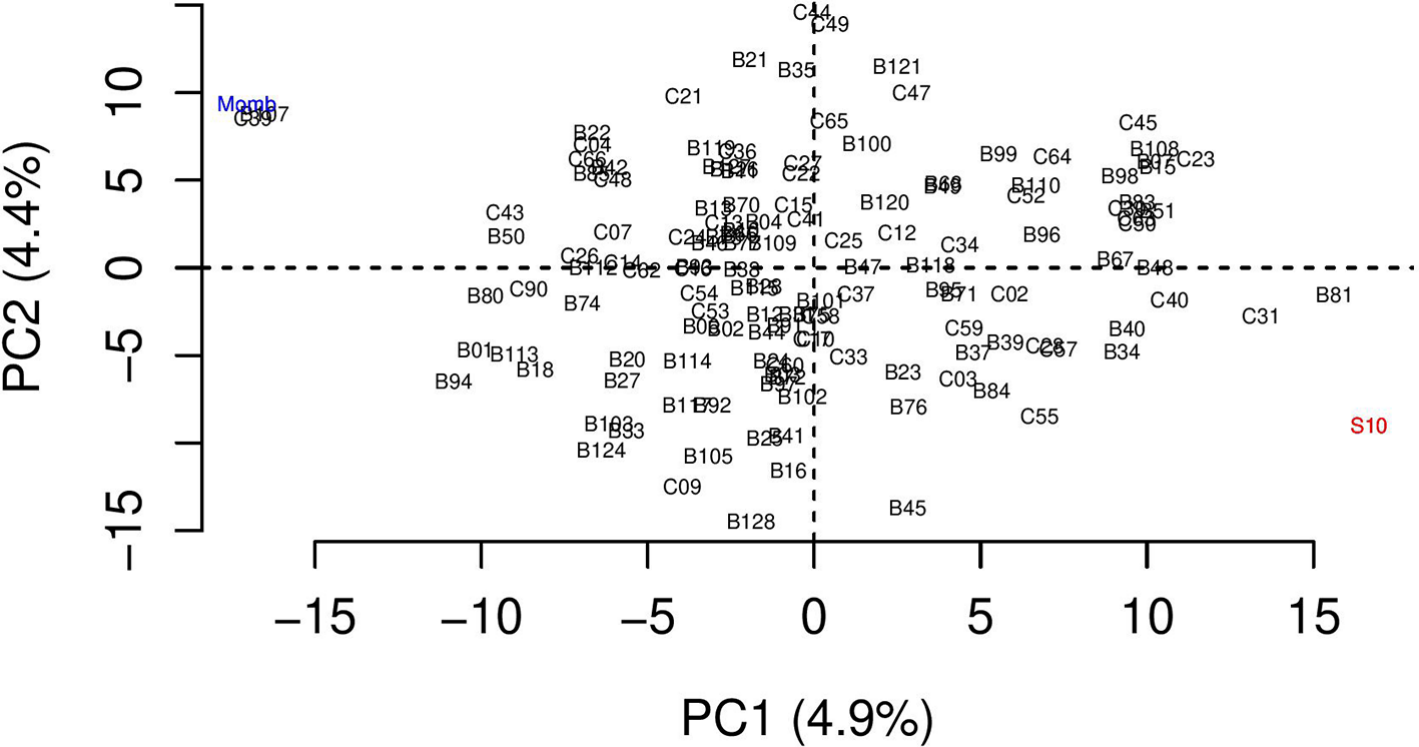
Principal component analysis representing the genetic diversity among the progeny and the parents of the mapping population of guinea grass (*Megathyrsus maximus*). The parents are highlighted in red (S10) and blue (cv. Mombaça).

A total of 23,619 SNPs were identified through alignment using the *P. virgatum* genome as the reference, and these presented a mean depth per individual of 247.8 (SD ± 121.7). After the genotypic analysis, 2,804 SNPs with an average allele depth greater than 60 were selected for further filtering. After allele dosage estimation, 275 SNPs were discarded, in addition to 15 monomorphic markers and four markers with missing data identified manually in R software. Using the updog package, we obtained the allele dosage information for 2,510 SNPs, considering an autotetraploid species (**Table 2**). Simplex (AAAA × AAAB, ABBB × BBBB) was the most commonly found configuration, with 1,654 markers, followed by the duplex (AAAA × AABB, AABB × BBBB) and double-simplex (AAAB × AAAB, ABBB × ABBB) configurations, with 334 and 226 SNPs, respectively. The simplex-duplex (AAAB × AABB) configuration was the most represented among the higher dosages, with 155 markers, and the duplex-simplex (AABB × ABBB) configuration was the least represented, with only six SNPs. Analysis with the polymapR package revealed that all offspring were 95% compatible with the parents. Two individuals (B107 and C39) were very genetically similar to the parent cv. Mombaça and were removed, one clone (C49) of individual C44 was also removed, and another clone (B127) similar to B126 was also excluded (**Figure 2**). We subsequently discarded another 37 SNPs with missing values and 1,151 duplicated SNPs. This filtering resulted in 132 genotyped offspring with 1,322 markers that were used at the beginning of the linkage analysis in TetraploidSNPMap software. In this step, incompatible markers with the parental allele dosages, SNPs with DR and markers showing SD were not considered. In addition, two-point analysis identified 149 SNPs as duplicated, and MDS analysis identified 27 outliers, which were then excluded. In total, 858 reliable SNPs were included in the linkage map. The apomictic parent, cv. Mombaça, presented 368 exclusive alleles; the sexual parent, S10, presented 275 exclusive alleles; and the two parents shared 215 alleles. TetraploidSNPMap software was used again to rank the 2,510 SNPs based on their expected segregation. The analysis resulted in a total of 114 SNPs with SD, 183 with NP and 243 with DR (**Table 2**).

**Table 2:** Distribution of SNP markers among genotype classes for a mapping population of guinea grass (*Megathyrsus maximus*).

Marker class	Genotype of the parents	Segregation ratio	Number	DR	NP	Distorted	Mapped
Null	AAAA x BBBB	0	0	0	0	0	0
Simplex	AAAA x AAAB, ABBB x BBBB	1:1	1,654	111	125	18	491
Duplex	AAAA x AABB, AABB x BBBB	1:4:1	334	0	58	40	136
Triplex	AAAA x ABBB, AAAB x BBBB	1:1	35	18	0	0	17
Double-Simplex	AAAB x AAAB, ABBB x ABBB	1:2:1	226	60	0	5	106
X-Double-Simplex	AAAB x ABBB	1:2:1	26	16	0	0	9
Simplex-Duplex	AAAB x AABB	1:5:5:1	155	38	0	9	72
Duplex-Simplex	AABB x ABBB	1:5:5:1	6	0	0	0	1
Double-Duplex	AABB x AABB	1:8:18:8:1	74	0	0	42	26
Total			2510	243	183	114	858

Significant distortion at (P < 0.001) and (P < 0.01) for simplex and higher-dosage markers. DR corresponds to double reduction, and NP indicates incompatible SNPs with the parental dosages. SNPs with missing data, outliers and duplicates were not considered.

### Linkage Map

**Figure 3:**
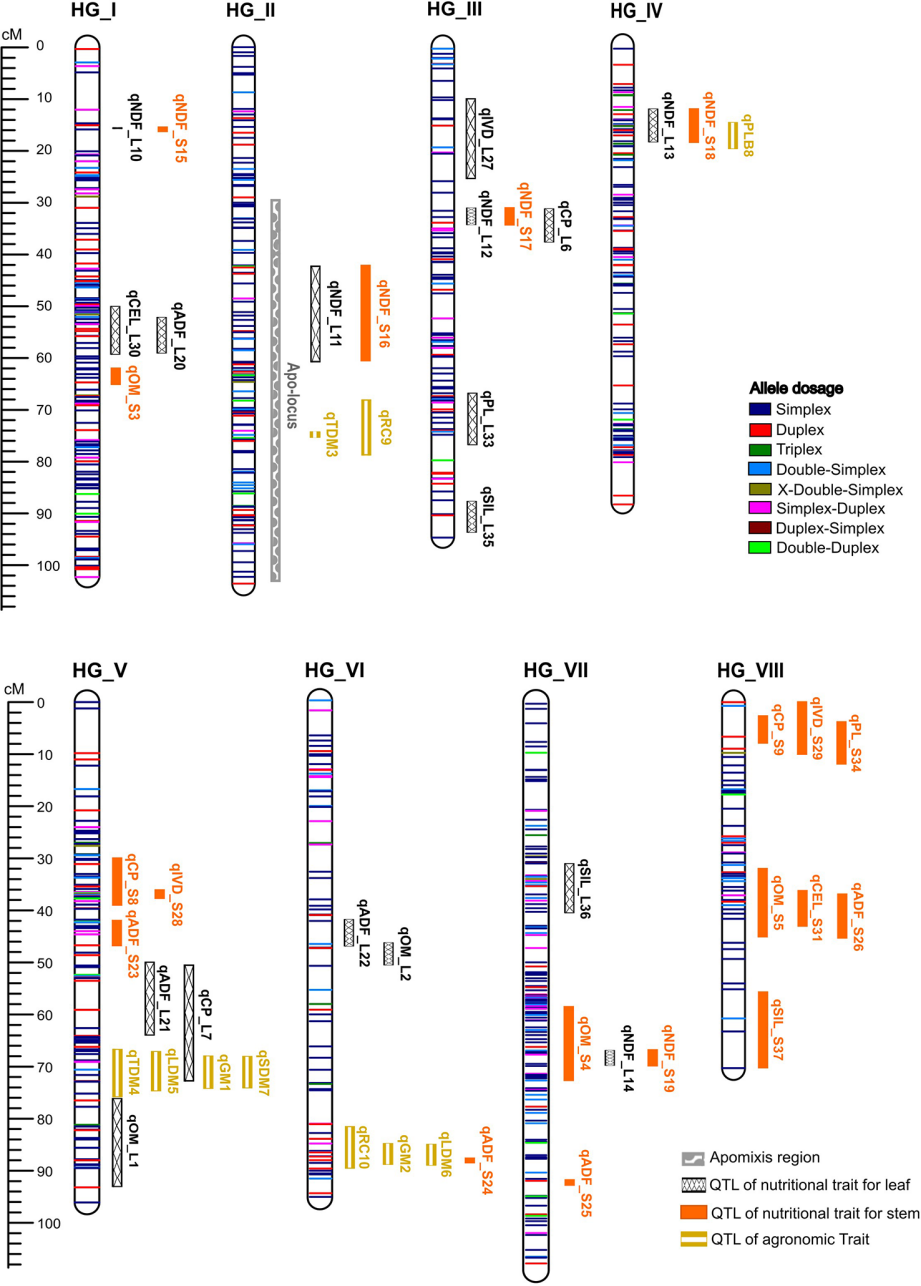
Linkage map constructed for guinea grass (*Megathyrsus maximus*) using SNPs with allele dosage information, including representation of intervals of the highest peaks of QTLs.

We constructed an integrated consensus linkage map consisting of 858 SNP markers distributed over 756.69 cM in eight HGs, with all possible allele dosage configurations for an autotetraploid species. (**Table 2**, **Figure 3** and **Supplementary Table 4**). Considering all integrated consensus HGs, an average density of ~1.13 SNPs/cM was obtained. The largest HG was VII, with 159 SNPs distributed over 108.573 cM, and the smallest HG was VIII, with 49 SNPs present over 70.05 cM. The interlocus intervals were relatively small, with a minimum value of 0.003 cM for the majority of the HGs (I, II, IV, VI and VII) and a maximum of 8.65 cM and 7.24 cM on HG V and HG I, respectively (**Table 3**). Among the markers, we identified approximately 30 double-duplex markers (AABB x AABB), which contained all types of doses for autotetraploid progenies (**Table 2**, **Figure 3** and **Supplementary Table 4**) (Hackett et al., 2014).

**Table 3:** Summary of the linkage map of guinea grass (*Megathyrsus maximus*) obtained with the S10 × cv. Mombaça population.

HG*	No. mapped SNPs	Length (cM)	Smallest interval (cM)	Longest interval (cM)
**I**	144	101.74	0.015	7.24
**II**	128	103.53	0.031	3.43
**III**	85	94.06	0.027	5.24
**IV**	115	87.66	0.003	6.34
**V**	104	96.08	0.008	8.65
**VI**	74	94.99	0.013	6.42
**VII**	159	108.57	0.009	5.63
**VIII**	49	70.05	0.072	6.93
**Total**	**858**	**756.69**		

*Homology group.

### Apospory Mapping

The mode of reproduction of 106 hybrids in the mapping population was determined and indicated that apospory had a segregation ratio of 1:1 based on a chi-square test (X² = 5.43, p ≥ 0.01), consistent with the model expected for monogenic inheritance. The apo-locus was mapped in the HG II at a peak position of 65 cM, with a high LOD score of 50.06 (**Supplementary Figure 1**). More than 80% of the phenotypic variation in the apomictic reproductive mode was explained, and the simple models best classified the apo-locus as a simplex genotype (BBBB x ABBB). As expected, this locus was linked to the apomictic parent, cv. Mombaça. Two SNP markers exclusive to this parent, namely, S_14_29023868 and S_10_48091934, were linked to the apo-locus at 0.8 cM (**Figure 3**).

### Agronomic Trait Mapping

Ten significant QTLs were mapped for GM, TDM, LDM, SDM, PLB, and RC, which were distributed in HGs II, IV, V and VI (**Table 4** and **Supplementary Figure 2**). Here, we will report the QTLs that were identified for the main traits targeted in the *M. maximus* breeding program. TDM was associated with two QTLs in HGs II (qTDM3) and V (qTDM4) with LOD scores of 3.4 and 4.4 and that explained 5.8 and 9.4% of the phenotypic variation, respectively. For LDM, we detected two QTLs in HGs V (qLDM5) and VI (qLDM6) with LOD scores of 3.9 and 3.0 and that explained 7.9 and 4.5% of the phenotypic variation, respectively. PLB was associated with one QTL in HG IV (qPLB8) with LOD score of 3.8 and that explained 6.8% of the phenotypic variation. We identified two QTLs for RC in HGs II (qRC9) and VI (qRC10) with LOD scores of 4.7 and 3.8 and that explained 10.4% and 6.3% of the phenotypic variation, respectively.

**Table 4:** QTLs identified for agronomic traits from the sexual genotype S10 and apomictic cv. Mombaça of guinea grass (*Megathyrsus maximus*).

Agronomic trait	QTL	HG	Position (cM)	LOD	R²	Parents
Green matter (GM)	qGM1	V	71.0	3.56	6.78	both
	qGM2	VI	87.0	2.91	4.29	‘Mombaça'
Total dry matter (TDM)	qTDM3	II	75.0	3.37	5.79	both
	qTDM4	V	70.0	4.41	9.40	S10
Leaf dry matter (LDM)	qLDM5	V	70.0	3.90	7.93	S10
	qLDM6	VI	88.0	2.97	4.53	‘Mombaça’
Stem dry matter (SDM)	qSDM7	V	71.0	3.73	7.24	‘Mombaça’
Percentage of leaf blade (PLB)	qPLB8	IV	17.0	3.81	6.84	both
Regrowth capacity (RC)	qRC9	II	75.0	4.73	10.37	S10
	qRC10	VI	87.0	3.77	6.25	‘Mombaça’

The simple models that best represented the QTLs for each agronomic trait were identified based on the lowest SIC values. The QTL with the greatest effect on TDM, which was present in HG VI, followed a duplex model (AAaa x aaaa) with an additive effect of the A allele from the S10 parent. The same model was verified for LDM with qLDM5, a QTL associated with the S10 parent, and qLDM6, a QTL associated with the cv. Mombaça parent. The QTL with the smallest effect for TDM and the single QTL for PLB followed the double-simplex model (Aaaa x Aaaa) with an additive effect of both parents. The two QTLs detected for RC followed the simplex model, with the S10 parent contributing the allele for qRC9 and the ‘Mombaça' parent contributing the allele for qRC10.

### Nutritional Trait Mapping

Thirty-six significant preliminary QTLs were identified for OM, CP, NDF, ADF, IVD, PL, CEL, and SIL for the leaf and stem. These QTLs were distributed in all HGs (**Table 5** and **Supplementary Figures 3**, **4**). For CP_L, two QTLs were detected in HGs III (qCP_L6) and V (qCP_L7) with LOD scores of 3.4 and 4.3 and that explained 5.1% and 9.7% of the phenotypic variation, respectively. For CP_S, two QTLs were detected in HGs V (qCP_S8) and VIII (qCP_S9) with LOD scores of 3.8 and 2.8 and that explained 8.4% and 3.2% of the phenotypic variation, respectively. For NDF_L and NDF_S, we detected five QTLs each, all at the same positions in HGs I (qNDF_L10/qNDF_S15), II (qNDF_L11/qNDF_S16), III (qNDF_L12/qNDF_S17), IV (qNDF_L13/qNDF_S18), and VII (qNDF_L14/qNDF_S19). The QTLs with the largest effect on NDF were found in HGs II and IV and were identified with LOD scores of 5.5 and 4.3, respectively; qNDF_L11/qNDF_S16 in HG II explained more phenotypic variation (12%). For the IVD_L trait, only one QTL was found in HG III (qIVD_L27) with LOD score of 3.8 and that explained 5.9% of the phenotypic variation. For IVD_S, two QTLs were obtained in HGs V (qIVD_S28) and VIII (qIVD_S29) with LOD scores of 3.6 and 3.9 and explaining 7.3 and 5.8% of the phenotypic variation, respectively. We detected a single QTL for PL_L located in HG III (qPL_L33) with LOD score of 3.9, and it explained 6.2% of the phenotypic variation. Additionally, PL_S was associated with only one QTL, which was identified in HG VIII (qPL_S34) with LOD score of 2.7 and explained 2.8% of the variation in the trait.

**Table 5:** QTLs identified for traits related to nutritional quality from the sexual genotype “S10” and apomictic cv. ‘Mombaça’ of guinea grass (*Megathyrsus maximus*).

Nutritional trait	QTL	HG	Position (cM)	LOD	R²	Parents
Leaf organic matter (OM_L)	qOM_L1	V	87.0	4.15	8.3	S10
	qOM_L2	VI	48.0	3.81	6.7	‘Mombaça’
Stem organic matter	qOM_S3	I	63.0	3.85	7.85	S10
(OM_S)	qOM_S4	VII	68.0	4.76	11.44	both
	qOM_S5	VIII	36.0	4.35	7.19	both
Leaf crude protein (CP_L)	qCP_L6	III	34.0	3.43	5.10	both
	qCP_L7	V	65.0	4.32	9.68	both
Stem crude protein (CP_S)	qCP_S8	V	33.0	3.84	8.40	‘Mombaça’
	qCP_S9	VIII	6.0	2.84	3.17	S10
Leaf neutral detergent fiber (NDF_L)	qNDF_L10	I	15.0	3.64	6.09	both
	qNDF_L11	II	48.0	5.49	12.08	both
	qNDF_L12	III	33.0	3.51	6.83	both
	qNDF_L13	IV	14.0	4.30	6.84	both
	qNDF_L14	VII	69.0	3.74	7.81	‘Mombaça’
Stem neutral detergent fiber	qNDF_S15	I	15.0	3.61	6.01	both
(NDF_S)	qNDF_S16	II	48.0	5.50	12.07	both
	qNDF_S17	III	33.0	3.5	6.81	both
	qNDF_S18	IV	14.0	4.31	6.87	both
	qNDF_S19	VII	69.0	3.75	7.85	‘Mombaça’
Leaf acid detergent fiber (ADF_L)	qADF_L20	I	57.0	3.98	8.84	S10
	qADF_L21	V	59.0	4.0	8.07	both
	qADF_L22	VI	45.0	3.08	2.46	both
Stem acid detergent fiber (ADF_S)	qADF_S23	V	45.0	3.48	6.21	‘Mombaça’
	qADF_S24	VI	88.0	2.86	4.05	‘Mombaça’
	qADF_S25	VII	93.0	3.41	5.90	Both
	qADF_S26	VIII	41.0	3.04	4.05	S10
Leaf in vitro digestibility of organic matter (IVD_L)	qIVD_L27	III	20.0	3.8	5.85	S10
Stem in vitro digestibility of organic matter (IVD_S)	qIVD_S28	V	37.0	3.55	7.31	‘Mombaça’
	qIVD_S29	VIII	6.0	3.91	5.84	
Leaf cellulose (CEL_L)	qCEL_L30	I	55.0	4.69	10.85	both
Stem cellulose (CEL_S)	qCEL_S31	VIII	37.0	3.02	3.37	both
Leaf permanganate lignin (PL_L)	qPL_L33	III	74.0	3.86	6.18	‘Mombaça’
Stem permanganate lignin (PL_S)	qPL_S34	VIII	7.0	2.7	2.78	both
Leaf silica (SIL_L)	qSIL_L35	III	94.0	3.70	5.68	‘Mombaça’
	qSIL_LF36	VII	36.0	4.63	10.63	S10
Stem silica (SIL_S)	qSIL_S37	VIII	61.0	2.96	4.23	both

According to the simple model analysis, the best model for the two QTLs of CP_L was the double-simplex model (Aaaa x Aaaa) with an additive effect of both parents. In contrast, qCP_S8 was best represented by a duplex model (AAaa x aaaa) with a dominant effect of cv. Mombaça. The CP_S9 QTL was represented by a double-simplex model with an additive effect of the S10 parent. The QTL with the greatest effect on NDF was best represented by the double-simplex model with an additive effect from both parents. For IVD_L, a simplex allele was verified in the sexual parent (Aaaa x aaaa). The same model was also observed for IVD_S and PL_L, with the A allele from the apomictic parent associated with the trait. The single QTL for PL_S was explained by a double-simplex model with a dominant effect from both parents.

### Search for Similarity in Aposporic and QTL Regions

Some genes that contain conserved protein domains were found in regions flanking the apo-locus located in HG II, such as the Spc97/Spc98 family of spindle pole body (SBP) components, whose proteins assist in the control of the microtubule network (Lin et al., 2015), and the inner centromere protein (ARK-binding region). These two genes play an important role in the segregation of chromosomes during cell division (Kirioukhova et al., 2011; Lin et al., 2015). In addition, a gene possibly involved in the aposporic reproductive mode was similar to somatic embryogenesis receptor-like kinase 1 (SERK1), which is part of a complex associated with the induction of embryo development (Albrecht et al., 2008).

A total of 23 regions were found due to the overlap of QTLs in common regions in the linkage map. In these regions, 55 different gene families from *P. virgatum*, *A. thaliana* and *O. sativa* were identified. Most of these genes may play important roles in cellular metabolism and may be associated with plant growth and development (**Table 6**). Further details about the locations of the candidate genes in their respective QTL regions are provided in **Supplementary Table 5**.

**Table 6:** Description and function of the genes identified in APOSPORY and QTL regions from linkage map of guinea grass.

HG	Region	QTL	Gene Description	Function	Reference
I	1	qNDF_L10	Rhomboid family protein	Root growth, floral development and fertility	Knopf et al. (2012)
		qNDF_S15			
I	2	qADF_L20	Phosphoenolpyruvate carboxylase	CO_2_ fixation in the cytoplasm	Toledo-Silva et al. (2013)
		qCEL_L30	Auxin efflux carrier family protein	Regulator of auxin efflux, differential growth and tropism	Friml et al. (2002)
			Inorganic H pyrophosphatase family protein	Regulation of plant proton‐pumping homeostasis	Primo et al. (2019)
			Vacuolar ATP synthase subunit A	Male gametophyte development and Golgi organization	Dettmer et al. (2005)
			Palmitoyltransferase TIP1	Stem cell or root hair and pollen tube growth	Hemsley et al. (2005)
			PINHEAD	Regulation of cell division and axis determinacy	Newman et al. (2002)
I	3	qOM_S3	Plant stearoyl-acyl-carrier-protein desaturase family protein	Regulation of oleic acid	Kachroo et al. (2007)
			Exocyst complex protein Exo70	Plant cell morphogenesis	Hála et al. (2008)
II	4	Apo-locus	Inner centromere protein (ARK-binding region)	Regulation of egg and central cell fate and differentiation	Kirioukhova et al. (2011)
			Somatic embryogenesis receptor-like kinase 1 (SERK)	Induction of somatic embryogenesis	Albrecht et al. (2008)
			Spc97 / Spc98 family of spindle pole body (SBP) component	Regulation of microtubule network	Lin et al. (2015)
II	5	qNDF_L11	RING/U-box domain-containing protein (XERICO)	Regulation of abscisic acid (ABA)	Ko et al. (2006)
		qNDF_S16	Wall-associated receptor kinase galacturonan-binding (GUB_WAK_bind)	Pathogen response and cell expansion	Kohorn and Kohorn (2012)
			Late embryogenesis abundant (LEA) protein-related	Seed maturation and tolerance to abiotic stress in plants	Zhang et al. (2014)
II	6	qTDM3	WRKY family transcription factor family protein	Pathogen defense, senescence and trichome development	Eulgem et al. (2000)
		qRC9	Eukaryotic elongation factor 5A-1	Regulation of cell division, cell growth, and cell death	Feng et al. (2007)
III	7	qIVD_L27	Glycosyltransferase	Biosynthesis of polysaccharides and glycoproteins	Hansen et al. (2012)
III	8	qCP_L6	Xyloglucan:xyloglucosyl transferase	Integral plasma membrane protein and wall‐loosening factor	Ndamukong et al. (2009)
		qNDF_L12	Xyloglucan endotransglucosylase/hydrolase	Cell wall construction	Yokoyama and Nishitani (2001)
		qNDF_S17			
III	9	qPL_L33	Protein thylakoid formation 1, chloroplastic	Vesicle-mediated thylakoid membrane biogenesis	Wang et al. (2004)
			Secoisolariciresinol dehydrogenase	Lignin biosynthesis	Davin and Lewis (2003)
			Gibberellin-regulated family protein	Reproductive development and regulation of growth	Hedden and Thomas (2016)
			NAD(P)-binding Rossmann-fold superfamily protein	Cinnamoyl-CoA reductase activity (lignin biosynthesis)	Pan et al. (2014)
III	10	qSIL_L35	Disease resistance protein RPS2 Stripe rust resistance protein	Immune signaling in response to pathogenic fungiImmune signaling in response to root-knot nematodes	Kourelis and van der Hoorn (2018) Dimkpa et al. (2016)
IV	11	qPLB8	Ent-copalyl diphosphate synthase	Gibberellin biosynthesis	Koksal et al. (2011)
		qNDF_L13	Phototropic-responsive NPH3 family protein	Phototropic signal response pathway	Roberts et al. (2011)
		qNDF_S18			
V	12	qCP_S8	Glucuronokinase	UDP-glucuronic acid synthesis (sugar metabolism)	Xiao et al. (2017)
		qIVD_S28	WRKY DNA-binding domain	Pathogen defense, senescence and trichome development	Eulgem et al. (2000)
			P-glycoprotein	Auxin transport	Geisler and Murphy (2006)
			Protein NRT1/ PTR family 6.2	Transporting different substrates, e.g., nitrate	Corratgé-Faillie and Lacombe (2017)
			Methyl esterase	Hydrolysis of auxin and jasmonic acid	Yang et al. (2008)
	13	qADF_S23	UDP-Glycosyltransferase superfamily protein	Mechanism of normal cell wall lignification	Lin et al. (2016)
V	14	qGM1	Early nodulin-like family protein	Cell differentiation and cell wall reorganization during nodulation	Mashiguchi et al. (2009)
		qTDM4	HEXOKINASE	Glucose regulation	Aguilera-Alvarado and Sánchez-Nieto (2017)
		qLDM5	Pectinesterase / Pectin methylesterase	Pectin structure	Pelloux et al. (2007)
		qSDM7	NAD-dependent malic enzyme (mitochondrial precursor)	Metabolism in mitochondria	Tronconi et al. (2008)
		qCP_L7	NADP-dependent malic enzyme (chloroplast precursor)	Metabolism in chloroplasts (C4 plants) or in cytosol	Tronconi et al. (2008)
		qADF_L21			
V	15	qOM_L1	Calcium-binding EF-hand family protein	Regulation of cellular and developmental processes	Day et al. (2002)
VI	16	qOM_L2	4-coumarate:CoA ligase 3	Lignin biosynthesis	Toledo-Silva et al. (2013)
		qADF_L22			
VI	17	qGM2	Galactinol-raffinose galactosyltransferase/stachyose synthetase	Desiccation protectant in seeds and transporter of sugar in phloem sap	Sengupta et al. (2015)
		qLDM6	Nodulin-like/major facilitator superfamily protein	Facilitators of water and ammonia transport	Routray et al. (2015)
		qRC10	Peroxidase superfamily protein	Lignin biosynthesis	Toledo-Silva et al. (2013)
		qADF_S24			
VII	18	qSIL_L36	FKBP-type peptidyl-prolyl cis-trans isomerase family protein	Control of cell proliferation and differentiation	Smyczynski et al. (2006)
VII	19	qOM_S4	Chloroplast envelope transporter	Ions transport	Höhner et al. (2016)
		qNDF_L14	Endo-1,4-beta-xylanase/glycosyl hydrolase family 10 protein	Xylan degradation	Suzuki et al. (2002)
		qNDF_S19	Alpha-galactosidase	Regulation of cell wall loosening and cell wall expansion	Chrost et al. (2007)
VII	20	qADF_S25	Lipoxygenase	Biosynthesis of polyunsaturated fatty acids	Bannenberg et al. (2008)
VIII	21	qCP_S9	3-oxoacyl-[acyl-carrier-protein] reductase	Fatty acid biosynthesis	Ding et al. (2015)
		qIVD_S29	Sugar transporter/spinster transmembrane protein	Transport of lipidic molecules	Niño-González et al. (2019)
		qPL_S34	Ethylene insensitive 3 family protein	Modulation of plant growth	Munné-Bosch et al. (2018)
			Glycine-rich cell wall structural transmembrane protein	Component of the cell walls of higher plants	Mousavi and Hotta (2005)
			NAD(P)-binding Rossmann-fold superfamily protein	Cinnamoyl-CoA reductase activity (lignin biosynthesis)	Pan et al. (2014)
VIII	22	qOM_S5	Sterol regulatory element-binding protein	Regulation of sterol biosynthesis	Seo et al. (2008)
		qADF_S26	Alpha carbonic anhydrase	C4 photosynthetic pathway	Toledo-Silva et al. (2013)
		qCEL_S31	Phosphatidylinositol-4-phosphate 5-kinase	Regulator of root hair tip growth	Kusano et al. (2008)
VIII	23	qSIL_S37	Pectinesterase	Pectin structure	Pelloux et al. (2007)

Among the putative genes, many participate in hormonal signaling pathways, such as auxin efflux carrier component in HG I region 2 (qADF_L20/qCEL_L30), which is related to the intercellular directionality of auxin (Friml et al., 2002); RING/U-box (XERICO) in HG II region 5 (qNDF_L11/ qNDF_S16), which is involved in the regulation of abscisic acid (ABA) (Ko et al., 2006); gibberellin-regulated family members (GAST1, GASR2, GASR3, and GASR9) in HG III region 9 (qPL_L33), which are associated with the regulation of gibberellic acid (GA) and ABA (Hedden and Thomas, 2016); ent-copalyl diphosphate synthase in HG IV region 11 (qNDF_L13/qNDF_S18/qPLB8), which plays a role in GA biosynthesis (Koksal et al., 2011); and ethylene insensitive 3 (EIN3) in HG VIII region 21 (qCP_S9/qIVD_S29/ qPL_S34), which is associated with cell growth and senescence processes (Munné-Bosch et al., 2018) (**Table 6** and **Supplementary Table 5**).

In addition, other genes with a role in plant physiology were also identified, such as the gene encoding the enzyme phosphoenolpyruvate carboxylase (PEPC) in HG I region 2 (qADF_L20/qCEL_L30), whose function is the catalysis of primary metabolic reactions in plants (Toledo-Silva et al., 2013). Glycosyltransferase was found in HG III region 7 (qIVD_L27) and is associated with the biosynthesis of polysaccharides and glycoproteins (Hansen et al., 2012). Pectinesterase/pectin methylesterase, which was detected in HG V region 14 (qGM1/qTDM4/qLDM5/qSDM7/qADF_L21/qCP_L7) and HG VIII region 23 (qSIL_S37) is related to cellular adhesion and stem elongation (Damm et al., 2016). In addition to these genes, we found a chloroplast envelope transporter in HG 7 region 19 (qOM_S4/qNDF_L14/qNDF_S19). Phosphatidylinositol-4-phosphate 5-kinase, which was present in HG VIII region 22 (qOM_S5/qADF_S26/qCEL_S31), is involved in coordinating plant growth (Kusano et al., 2008).

Many putative candidate genes present in lignan biosynthesis pathways were verified in some regions of QTLs. Glycosyl hydrolase family 16 was located in HG III region 8 (qCP_L6/qNDF_L12/qNDF_S17), and the genes associated with secoisolariciresinol dehydrogenase and the NAD(P)-binding Rossmann-fold superfamily were located in region 9 (qPL_L33). UDP-Glycosyltransferase superfamily protein was present in HG V region 13 (qADF_S23). The 4-coumarate:CoA ligase 3 gene was obtained in HG VI region 16 (qOM_L2/qADF_L22). The gene encoding NAD(P)-binding Rossmann-fold superfamily was also detected in HG VIII region 21 (qCP_S9/qIVD_S29/ qPL_S34).

## Discussion

### Linkage Map

We obtained a satisfactory representative linkage map for *M. maximus*, with a large genetic distance observed between the parents, namely, cv. Mombaça. (**Figure 2**, quadrant I) and S10 (**Figure 2**, quadrant IV). This genetic distance and the distribution of hybrids can be visualized in the PCA performed with the allele dosage information of all individuals (**Figure 2**). The crossing of contrasting parents makes it possible to check the recombination frequency between loci, which is a fundamental principle for observing the segregation of traits in hybrids and promoting the detection of QTLs (Luo et al., 2000).

Because *M. maximus* does not yet have a sequenced genome, we aligned our GBS reads to the allotetraploid genome of *P. virgatum*, which is a species closely phylogenetically related to *M. maximus* (Burke et al., 2016), since *M. maximus* previously belonged to the *Panicum* genus. This genetic proximity was confirmed based on the consistent distribution of the markers in the genetic map (**Figure 3** and **Supplementary Table 4**). In addition, considering our tetraploid mapping population, the use of a tetraploid genome as reference might be more informative than the use of diploid genomes because there is a greater possibility of similar chromosomal rearrangements between these species (Daverdin et al., 2015). However, the possibility of using the genome of *P. virgatum* as reference does not preclude the need for a sequenced genome of *M. maximus*, mainly due to the possibility of identifying exclusive markers of the species, and our linkage map can contribute to the assembly of this reference genome.

After allele depth estimation, only 10% of markers were retained for the next analysis, in which we prioritized a minimum average allele depth of 60 reads to suppress the probable overestimation of allele bias due to our population size. Gerard et al. (2018) recommend that more than 25 reads be used to obtain a strong correlation of true genotypes under high levels of bias and overdispersion, emphasizing that read depth requirements should be based on how many individuals are included in the study. Of the genetic mapping studies involving tropical forage grasses, only one reported the use of SNP markers with allele dosage, and in the study, a minimum overall depth of 25 reads was considered in a biparental progeny of *U. decumbens* containing 217 F_1_ hybrids (Ferreira et al., 2019).

Updated statistical models and the recent genome sequencing technologies promoted advances in the knowledge of the genetics of polyploid organisms, as demonstrated by the comparison of our genetic map for *M. maximus* with the first map for this species published over ten years ago by Ebina et al. (2005). The first map contained 360 dominant markers obtained with amplified fragment length polymorphism (AFLP) and random amplified polymorphic DNA (RAPD) techniques that segregated at a 1:1 ratio and was obtained from an apomictic cultivar used for genetic breeding in Japan. These markers were distributed in 39 linkage groups, which is greater than the number expected for this species (2n = 4x = 32). In this context, the use of SNPs as codominant markers and their quantitative analysis allowed many gains in our map, such as coverage of many regions of quantitative traits important to breeders and the genetic effects that influence these traits, as well as alleles of the parents that were determinant of the characters of the hybrid.

In addition, the strategy of greater refinement of SNPs adopted after estimating allele dosage using two programs of mapping prevented overinflation among loci. We detected some markers with SD (**Table 2**), but we chose not to use them, aiming to increase the probability of obtaining an exact distance between markers in the HGs. Since genotyping errors are probable, a large amount of missing data and a large number of distorted loci may promote the expansion of linkage maps as well as overestimate the recombination fractions and limit the accuracy of the mapping (Cartwright et al., 2007; Gerard et al., 2018). However, SD is a phenomenon commonly found in the genome, and some linkage maps contain distorted markers, including those of grasses such as pearl millet (Sehgal et al., 2012) and napiergrass (Paudel et al., 2018). Therefore, greater knowledge of the occurrence and genetic causes of SD in plants is important for inferring which genes are kept together or separated by SD (Zhu et al., 2006; Anhalt et al., 2008). Thus, future studies might aggregate information on the structure of loci with SD in the genome of *M. maximus*.

Most linkage maps in grasses include only single-dose markers with segregation ratios of 1:1 and 3:1; thus, the heterozygous classes were grouped with one of the homozygous classes, resulting in a loss of information (Bourke et al., 2018b). The use of linkage maps with simplex SNPs for most linkage groups and a high density of higher-dosage markers provides greater confidence in the modeling of allelic effects of QTLs (Hackett et al., 2014). The markers present in our map were mostly simplex, while higher-dosage markers comprised approximately 28.4% of the map, as shown in **Table 2**. Similar values were also verified in the genetic map of *U. decumbens* (Ferreira et al., 2019), probably due to the complexity of scoring and analyzing these types of markers. Our map presented a greater number of alleles exclusive to the apomictic parent than sexual parent, as observed in the previous map of *M. maximus* (Ebina et al., 2005) and in the genetic maps of other forage grasses (Worthington et al., 2016; Ferreira et al., 2019). This difference is likely due to the origin of the genotypes: "Mombaça" and S10 have natural tetraploid genomes, but S10 was obtained from a sexual x apomictic cross of an original diploid sexual plant that was duplicated with colchicine.

Our linkage map was sufficient for the identification of an oligogenic trait with a genomic region responsible for 80% of the phenotypic variance and polygenic traits. Therefore, our map will also be useful for the detection of other important characteristics in *M. maximus* and can contribute to the assembly of the genome of this species, as well as to studies about the biology and evolution of other phylogenetically closely related tropical forage grasses, such as those in the *Urochloa* and *Paspalum* genera.

### Double Reduction in Guinea Grass

We reported for the first time the occurrence of DR in *M. maximus*. Autotetraploids may undergo this type of segregation when in multivalent pairing, two pairs of chromatids pass to the same pole in anaphase I of meiosis (Haynes and Douches, 1993). In guinea grass, irregular chromosome segregation has already been verified in cytogenetic analysis in hybrids and parents from the breeding program of the Embrapa Beef Cattle (Pessim et al., 2010; Pessim et al., 2015). The distribution of the markers with DR between the dosage types was proportional to the number of SNPs with each configuration. DR has been extensively studied using SNPs with dosage data in autotetraploid linkage maps in potato (Hackett et al., 2017; Bourke et al., 2018a). Approximately 6% of markers in potato have DR (Bourke et al., 2015), corroborating our results (9.68%). Despite some studies suggesting that DR should be included in genetic map construction and in QTL analysis (Li et al., 2010), other studies verified that such markers exert only minor positive effects on the power and accuracy of mapping analysis using single-dose markers (Bourke et al., 2015) and higher-dose markers (Bourke et al., 2016). In addition, statistical models have been created to include DR in linkage mapping (Huang et al., 2019), but no software currently implements them.

The occurrence of DR in *M. maximus* has many implications for breeding programs, being that the effects of DR and how to handle them have been the targets of several studies (Luo et al., 2000; Xu et al., 2013; Layman and Busch, 2018). This type of segregation exposes alleles located in distal regions of the chromosomes to homozygosis and thus is effective in eliminating the lethal alleles in a population (Butruille and Boiteux, 2000). A low rate of DR is sufficient to considerably reduce the equilibrium frequency of a deleterious allele at one locus (Luo et al., 2006). As an alternative, DR could be used to accelerate the accumulation of favorable rare alleles through marker-assisted selection (MAS) (Bourke et al., 2015). In addition, it is possible to obtain genotypes with loci having a higher homozygosis rate for use in specific crosses (Bourke et al., 2015). In this context, more detailed molecular study could elucidate the influence of DR on the phenotypes of hybrids of our study species.

### Apospory Mapping and the Search for Gene Similarity

A chi-square test (X² = 5.43, p ≥ 0.01) performed for qualitative analysis of the reproductive mode of the 106 hybrids followed the Mendelian inheritance model, corroborating the results obtained with progeny tests in guinea grass performed by Savidan (1980) and Savidan (1981), in which sexual x apomictic progenies exhibited a 1:1 ratio that could be explained by an Aaaa genotype for apomictic because the aposporous apomixis of *M. maximus* is dominant over sexuality. We also proved this finding through SNP markers. Other studies of grasses such as *Pennisetum* (Akiyama et al., 2011), *Paspalum* (Martínez et al., 2003) and *Urochloa* (Valle et al., 1994; Vigna et al., 2016) also verified this segregation for the reproductive mode. Evidence suggests that this locus is present in a conserved region of the plant genome; however, further molecular genomic studies on aposporous apomixis in forage grasses are needed because the recent studies have led to other hypotheses, such as a possible influence of epigenetics (Kumar, 2017). Interestingly, more advanced studies on apomixis in *M. maximus* genotypes from a germplasm bank of India reported the decoupling of apomixis into three components, namely, apospory, parthenogenesis and pseudogamous-endosperm. These components are three distinct genetic determinants that determine an individual as apomictic, and recombination might occur among these components, supporting the hypothesis that more major genes are involved in apomixis control (Kaushal et al., 2008; Kaushal et al., 2019). This decoupling in neo-apomictics might be the target of an adaptive mechanism to maintain variability through hybridization and could be beneficial to the breeding program (Kaushal et al., 2019).

The aposporous region in *M. maximus* was previously mapped (Ebina et al., 2005; Bluma-Marques et al., 2014), and similar to our results, no markers were in perfect linkage with the region. Nonetheless, we mapped markers at a shorter distance (0.8 cM) from the apo-locus (**Figure 3**). Genetic markers linked to apomixis have been sought in other tropical forage grasses (Vigna et al., 2016; Worthington et al., 2016; Worthington et al., 2019), aiming at the efficient and rapid identification of the reproductive mode of progenies. Once identified, such markers may be transferred among forage grasses, based on evidence of conservation of the ASGR. Thus, markers near the apospory region that were identified in our map may be validated and useful for the breeding program of this species.

In addition, we observed that the markers closer to the peak region of the apo-locus were in a genomic region of *P. virgatum* similar to a region of the genome of *A. thaliana* that contains the SERK1 gene. This gene is involved in the signaling pathway active during zygotic and somatic embryogenesis in *A. thaliana*, and its overexpression increases the efficiency of somatic embryogenesis initiation (Hecht et al., 2001). In nucellar cells of apomictic genotypes of *Poa pratensis*, the SERK gene is involved in embryo sac development (Albertini et al., 2005). Recently, SERK was reported in *Brachypodium distachyon* grass as having a domain conserved among monocots and plays a prominent role in apomixis (Oliveira et al., 2017). Scarce studies have investigated the genes involved in the regulation of reproductive events in guinea grass, and these previous studies mainly utilized of transcriptome data (Toledo-Silva et al., 2013; Radhakrishna et al., 2018).

### QTLs for Agronomic and Nutritional Traits

A QTL mapping approach is required for the characterization of the genetic architecture of traits. In our study, QTL analysis of autotetraploid progeny was performed using interval mapping (IM) of markers with allele dosage. This same methodology was successfully applied in QTL mapping in signalgrass, another important forage grass (Ferreira et al., 2019). The multiple interval mapping (MIM) method was recently implemented in polyploids and is a new alternative for other mapping studies using data with allele dosage information (Pereira et al., 2019). The mapping method used here considered only the peak with the largest effect as a QTL, but it is worth mentioning that peaks were present near the peak QTL for all agronomic traits (**Supplementary Figure 2**).

QTLs associated with important agronomic traits were mapped in HGs I, III, VII and VIII, with the phenotypic variation explained ranging from 4.3% to 10.4% (**Table 4**). Because *M. maximus* is undergoing a domestication process, the crop can still be greatly improved by the selection of large-effect QTLs. Therefore, QTL qRC9, located at 75 cM in HG II, which explained 10.3% of the phenotypic variation in RC (**Supplementary Figure 2**, HG_2(B)) and had a predominant additive effect of the female progenitor S10, may be a candidate for the marker-assisted selection program of guinea grass.

We found more than one QTL for TDM, LDM, and RC, again supporting the hypothesis of complex genetic control. Conversely, we found only one QTL for PLB in both parents. TDM and LDM showed high broad-sense heritability (<0.5), followed by RC (0.3) and PLB (0.1), as shown in **Table 1**. Higher heritability values (<0.85) for LDM and RC and a value for PLB above 0.4 were recently reported in *M. maximus* (Lara et al., 2019), using the generalized heritability formula (Cullis et al., 2006). For this species, a greater amount (g/plant) of TDM and LDM in the progenies has been associated with considerable heritability from the most productive parents (Braz et al., 2013; Braz et al., 2017). Matias et al. (2019) also verified the same pattern in interspecific hybrids from *Urochloa* spp., another genus adapted to tropical conditions. The intermediate heritability of RC and the QTLs associated with this trait that were detected in both parents resulted in hybrids with a good capacity for regrowth. This trait is also considered fundamental in forage grasses because it is directly related to the persistence of the forage after defoliation (Jank et al., 2011).

The negative correlation between PLB and SDM was expected (**Figure 1**), and Braz et al. (2017) verified the higher PLB values in the experiment of this progeny. PLB is related to plant structure, and plants with a high percentage of leaves are desirable because this trait is related to higher forage quality. A higher PLB was observed in the male parent, cv. Mombaça, which is often used as a check in experiments. Since its release in the 1990s, along with cv. Tanzania, cv. Mombaça has promoted pasture intensification in the country due to its very high productivity and forage quality (Jank et al., 2014). Progenies whose female parent is S10 generally also present good yield (Resende et al., 2004). Breeding programs target these traits in search of superior genotypes with greater foliar mass and a higher percentage of leaves due to the higher digestibility of leaves than of stems for animals. Thus, forage breeding is not restricted to the obtaining of more productive plants; it also contributes to greater efficiency in their transformation into animal production (Valle et al., 2009).

Significant and positive correlations among the traits GM, TDM, SDM, LDM, and RC corroborated the positions of QTLs associated with agronomic traits in the linkage map (**Figures 1** and **3**). The traits GM and LDM exhibited high heritability (**Table 1**) and strong positive correlation (**Figure 1**), which suggests that genetic improvements in these traits could be achieved by selective phenotyping for only one of these traits, without the need to do separation of plant part. We detected qTDM3 and qRC9 in a common region in HG II; qGM1, qTDM4, qLDM5, and qSDM7 in the same region in HG V; and qGM2, qLDM6, and qRC10 in part of a common region in HG VI. Each region containing several QTLs for different traits suggests the occurrence of four QTL hotspots. Interestingly, QTLs for PLB were not detected in any region with other agronomic traits, and a negative correlation has also been observed; however, a positive correlation with NDF was verified, and such a QTL was detected in a similar region with qNDF_L13 and qNDF_S18 in HG IV (**Figure 3**), suggesting a fifth QTL hotspot.

Clustering of QTLs for genetically correlated traits in the same or adjacent regions of HGs in several organisms may be due to physical linkage, pleiotropy or natural selection for coadapted traits (Studer and Doebley, 2011; Wu et al., 2015). We have taken the first step in the identification of loci that lead to these trait correlations and the degree to which these patterns affect productivity in *M. maximus*. QTLs colocated in the same region of HGs for agronomic traits have also been identified in some grasses (Fang et al., 2016; Sartie et al., 2018).

All HGs contained QTLs related to nutritional traits, with those related to the leaf and stem being found mainly in HG III and HG VIII, respectively. Again, some traits had more than one peak that could not be considered due to the methodology adopted (**Supplementary Figures 3**, **4**). The phenotypic variance explained by these QTLs ranged between 2.5% (qADF_L22) and 12.1% (qNDF_L11 and qNDF_S16). Interestingly, both parents contributed alleles for most of the identified QTLs, providing evidence that the genotypes have high nutritional quality.

The selection of a superior genotype of perennial tropical forages is based on the analysis of traits through repeated measures over a number of harvests, seasons, and years (Fernandes et al., 2017). The nutritional QTLs found in our study need further investigation because only one harvest was analyzed and interactions could not be measured. In a selection study of *U. decumbens* and *U. humidicola*, Figueiredo et al. (2019) identified a significant Genotype x Harvest Interaction (GHI) effect (p<0.05) for different agronomic and nutritional traits, which reflect differences in the relative performances of genotypes across harvests. GHI also has been reported as significant in *M. maximus*, and the average number of harvests needed for a reliable selection of nutritional traits, such as OM and CP, would be 3 and 5 at accuracy levels of 0.80 and 0.85, respectively (Fernandes et al., 2017). Thus, GHI directly affects the selection of the genotypes and further studies with more harvests are needed to consolidate our results about putative QTLs to nutritional traits.

The heritability of the nutritional traits varied from low (0.06), obtained for SIL_S, to moderate (0.32), obtained for OM_L. This result also reinforces the necessity of more harvests for the efficient selection of superior genotypes for nutritional traits (Fernandes et al., 2017). Traits IVD_L and IVD_S presented the same standard as the crude protein, with the H² of the stem being higher than that of the leaf (**Table 1**). Historically, cv. Mombaça has stood out due to its high productivity, but with slightly lower values for forage quality when compared to ‘Tanzania', and in biparental crosses, the hybrids obtained from “Mombaça” also presented these features (Braz et al., 2017). This finding was corroborated by the heritability verified above in our study of agronomic and nutritional traits.

The nutritional quality of forage grasses is important in several aspects and is directly related to the production of meat and milk. *M. maximus* shows a high value of CP compared to other tropical forages. However, the values for lignin and fiber are expected to be low because lignin hampers the enzymatic hydrolysis of cellulose and hemicellulose and, thus, the digestion of the cell wall of the leaf tissue and the stem (Jung and Allen, 1995). The relationship between the biomasses of leaves and stems is important due to its effects on nutritional value and voluntary consumption by animals. The NDF is associated with fibrous fractions and with voluntary consumption. The fractions that are not digested by the animal take up space in the digestive tract, impairing the digestion and consumption of dry matter (Euclides et al., 1999).

NDF_L and NDF_S showed a strong positive correlation, and their respective QTLs were in the same regions in the HGs. In the linkage map, qCEL_L30 and qADF_L20 QTLs were in the same region, and qCEL_S31 and qADF_S26 were both located in HG VIII and were significantly correlated (**Figure 1**). The traits CP and IVD showed a weak positive correlation, but the qCP_S8 and qIVD_S28 QTLs were present in the same region of HG V, and qCP_S9 and qIVD_S29 were identified in the same region of HG VIII. Interestingly, qPL_S34 was verified in HG VIII in the same region as CP and IVD QTLs, and qADF_L21 shared a similar region with qCP_L7 in HG V. In addition, qCP_L7 extended to QTLs related to agronomic traits (qGM1, qTDM4, qLDM5 and qSDM7). A total of 8 probable QTL hotspots have been identified, supporting the need for further studies in search of a specific gene controlling all these traits or several genes acting together.

Notably, in the progenies of *M. maximus* from lower-yielding parents, the nutritional value is generally higher. With the identified QTLs, more in-depth studies of this correlation will be possible. In addition, QTLs related to forage quality have not been found in other important tropical species, such as *Urochloa* spp. Therefore, our results can contribute to the search for important genomic regions in other forage species.

### Search for Similarity in QTL Regions

The search for putative candidate genes was based on all 23 QTL regions. Generally, the same gene families from *A. thaliana*, *O. sativa*, and *P. virgatum* were identified for a common QTL region. These genes are also found in the literature and are described in **Table 6** and **Supplementary Table 5**.

Exploration of genes involved in plant growth and development, especially those related to hormone regulation, is crucial in forage grass breeding programs. Interestingly, the gibberellin family (GA_S_) was identified in HG III region 9 (qPL_L33), whose QTL is related to lignin, a complex phenolic polymer deposited in the secondary cell wall of all vascular plants (Zhao, 2016). The interrelations between cell wall components cause cellulose and lignin to be codependent, a normal cellulose deposition pattern may be necessary for lignin assembly, and alterations of lignin content may lead to changes in the cell orientation of cellulose fibrils and, consequently, in digestibility (Anderson et al., 2015; Liu et al., 2016). GAs promote biochemical, physiological and anatomical plant changes (Hedden and Thomas, 2016). The induction of cellulose synthesis by GAs promotes the release of secondary regulators of cell wall proteins and, consequently, can boost lignin deposition and increase lignin content (Zhao, 2016). GA_S_ at increased light levels have been shown to promote cell wall thickness and increase lignin deposition in xylem fibers (Falcioni et al., 2018).

Other important genes identified are associated with pectinesterase/pectin methylesterase and were present in HG V—region 14 (qGM1/qTDM4/qLDM5/qSDM7/qADF_L21/qCP_L7) and HG VIII—region 23 (qSIL_S37), which also contained agronomic QTLs. Pectinesterase is responsible for the hydrolyzation of pectin, the major component of cell walls (Pelloux et al., 2007). In addition, this enzyme is involved in developmental processes such as stem elongation in *A. thaliana* (Damm et al., 2016) and in *B. distachyon* grass (Feng et al., 2015). However, more in-depth research should be performed to ensure an understanding of the signaling pathways of these genes and make this understanding applicable to tropical forage breeding programs.

In conclusion, the present study produced a high-resolution linkage map with allele dosage information obtained from a full-sib progeny of *M. maximus* with high genetic variability. Even without the availability of a sequenced genome for this species, the approach adopted for the construction of our map was sufficient to detect many QTLs associated with agronomic and nutritional traits that are important for forage breeding. Our genetic map also allowed us to map the apo-locus to a single linkage group and provided a more up-to-date study of the mode of reproduction of *M. maximus*. The knowledge about the genetics of these traits that we obtained is the first step in discovering genes involved in relevant biological processes as well as understanding the genetic architecture of relevant traits in this species.

## Data Availability Statement

The datasets generated for this study can be found in the NCBI/PRJNA563938.

## Author Contributions

AG, LJ, and AS conceived and designed the experiments. MS and LJ conducted the field experiments. TD, RF, AM, AP, and FO performed the laboratory experiments. TD, RF, LL, AM, AA-P, and FO analyzed the data. TD, RF, and LL wrote the manuscript. All authors read and approved the manuscript.

## Funding

This work was supported by grants from the Fundação de Amparo à Pesquisa do Estado de São Paulo (FAPESP 2008/52197-4), the Conselho Nacional de Desenvolvimento Científico e Tecnológico (CNPQ), the Coordenação de Aperfeiçoamento de Pessoal de Nível Superior (CAPES—Computational Biology Program), Embrapa and UNIPASTO. TD received PhD fellowships from the CAPES Computational Biology Program and an MSc fellowship from FAPESP (2017/17969-5) and the CAPES Computational Biology Program. RF, FO, and AP received postdoctoral fellowships from FAPESP (2018/19219-6, 2018/18527-9 and 2018/00036-9, respectively). LL received a postdoctoral fellowship from the CAPES Computational Biology Program. AG and AS were recipients of a Research Fellowship, and LJ was recipient of a Technological Development scholarship, all received from the Conselho Nacional de Desenvolvimento Científico e Tecnológico (CNPq).

## Conflict of Interest

The authors declare that the research was conducted in the absence of any commercial or financial relationships that could be construed as a potential conflict of interest.

## Supplementary Material

Figure S1 Identification of the apospory region position (cM) in HG II from guinea grass mapping population. Dotted line indicate the LOD thresholds of 90% and 95% obtained after the permutation tests.

Figure S2 Interval mapping (IM) for agronomic traits from the guinea grass (Megathyrsus maximus) population in HGs II, IV, V and VI. Agronomic traits: green matter (GM), total dry matter (TDM), leaf dry matter (LDM), stem dry matter (SDM), regrowth capacity (RC) and percentage of leaf blade (PLB). Dotted lines indicate the LOD thresholds of 90% and 95% obtained after the permutation tests.

Figure S3 Interval mapping (IM) for forage quality from the guinea grass (Megathyrsus maximus) population in HGs I to IV. Nutritional quality traits for the leaf (L) and/or stem (S): organic matter (OM_S), crude protein (CP_L), in vitro digestibility of organic matter (IVD_L), neutral detergent fiber (NDF_L and NDF_S), acid detergent fiber (ADF_L), cellulose (CEL_L), silica (SIL_L), and permanganate lignin (PL_L). Dotted lines indicate the LOD thresholds of 90% and 95% obtained after the permutation tests.

Figure S4 Interval mapping (IM) for forage quality from the guinea grass (Megathyrsus maximus) population in HGs V to VIII. Nutritional quality traits for the leaf (L) and/or stem (S): organic matter (OM_L and OM_S), crude (CP_L and CP_S), in vitro digestibility of organic matter (IVD_S), neutral detergent fiber (NDF_L and NDF_S), acid detergent fiber (ADF_L and ADF_S), cellulose (CEL_S), silica (SIL_L and SIL_S), and permanganate lignin (PL_S). Dotted lines indicate the LOD thresholds of 90% and 95% obtained after the permutation tests.
