# Supplementary figures and images for: High-Resolution Linkage Map With Allele Dosage Allows the Identification of Regions Governing Complex Traits and Apospory in Guinea Grass (Megathyrsus maximus)
Source: Front Plant Sci. 2020 Feb 26;11:15. doi: 10.3389/fpls.2020.00015 (PMC7054243; doi:10.3389/fpls.2020.00015)

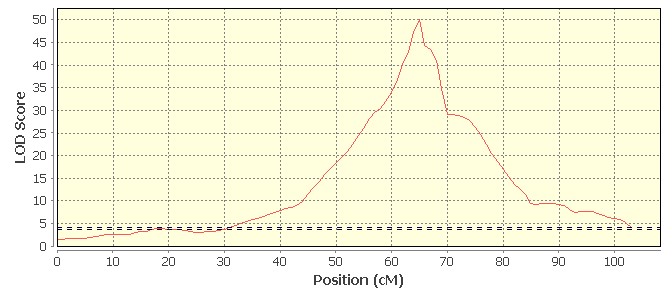

Supplement: Figure S1 — Identification of the apospory region position (cM) in HG II from guinea grass mapping population. Dotted line indicate the LOD thresholds of 90% and 95% obtained after the permutation tests. [file Image_1.jpeg]

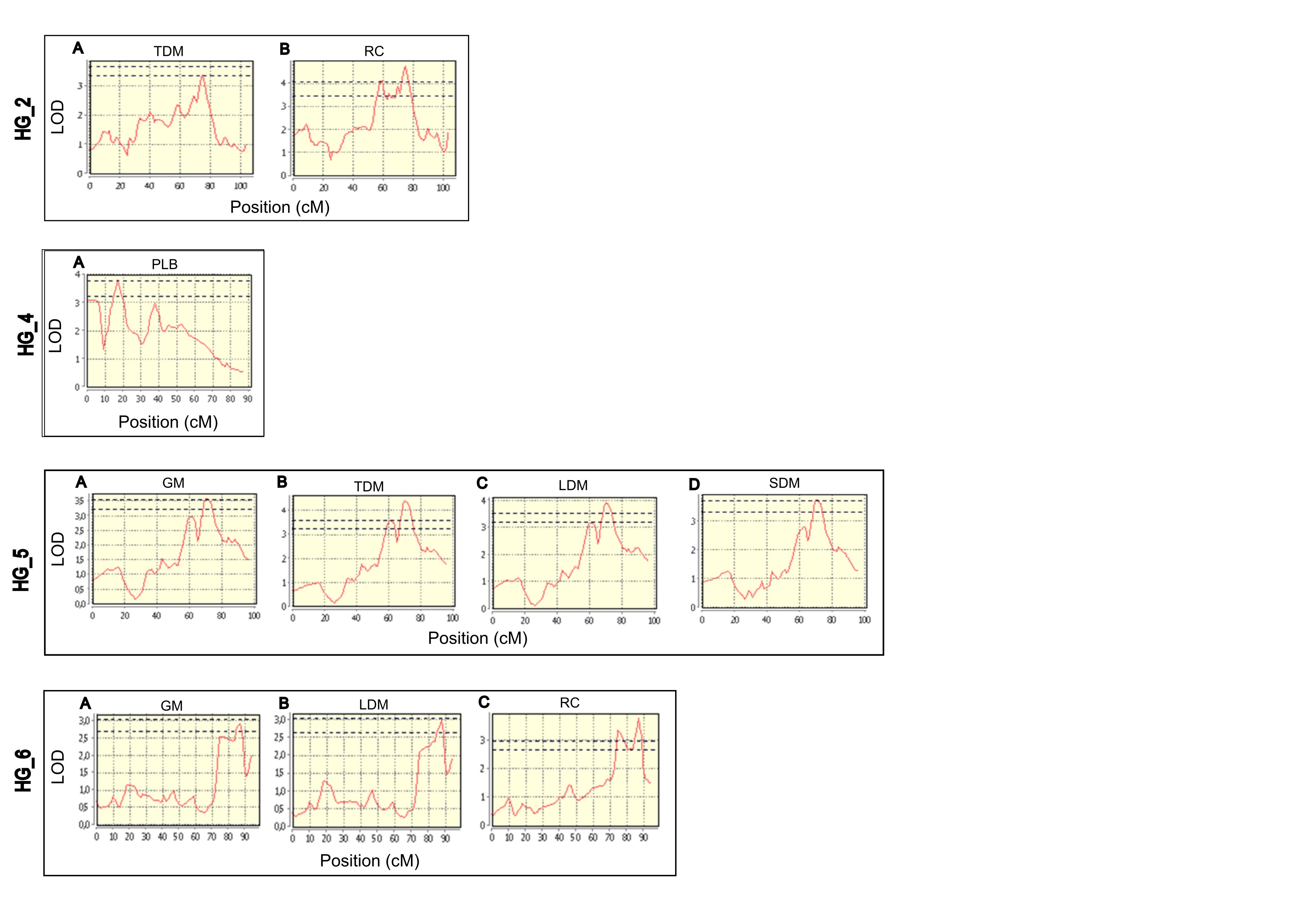

Supplement: Figure S2 — Interval mapping (IM) for agronomic traits from the guinea grass (Megathyrsus maximus) population in HGs II, IV, V and VI. Agronomic traits: green matter (GM), total dry matter (TDM), leaf dry matter (LDM), stem dry matter (SDM), regrowth capacity (RC) and percentage of leaf blade (PLB). Dotted lines indicate the LOD thresholds of 90% and 95% obtained after the permutation tests. [file Image_2.jpeg]

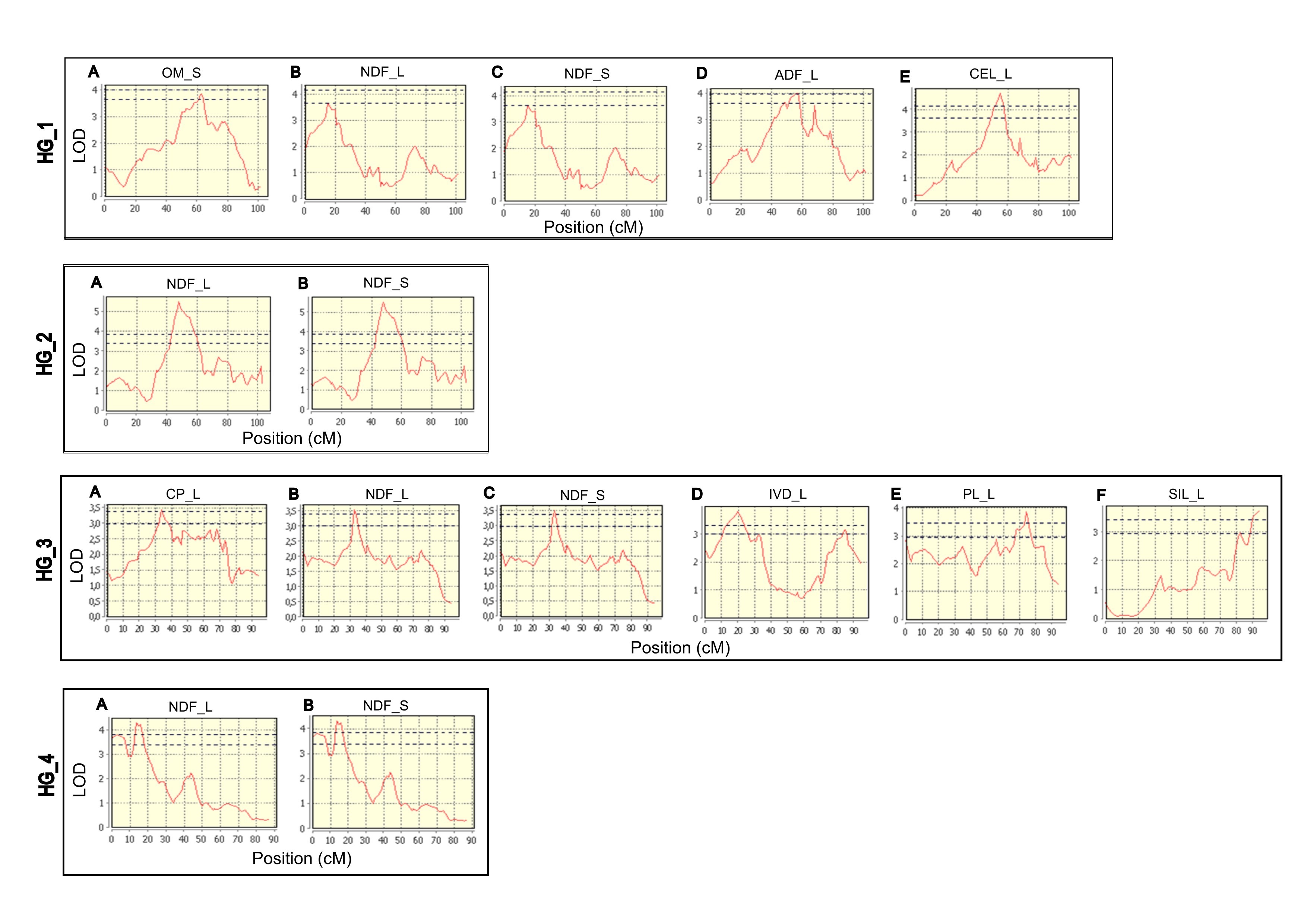

Supplement: Figure S3 — Interval mapping (IM) for forage quality from the guinea grass (Megathyrsus maximus) population in HGs I to IV. Nutritional quality traits for the leaf (L) and/or stem (S): organic matter (OM_S), crude protein (CP_L), in vitro digestibility of organic matter (IVD_L), neutral detergent fiber (NDF_L and NDF_S), acid detergent fiber (ADF_L), cellulose (CEL_L), silica (SIL_L), and permanganate lignin (PL_L). Dotted lines indicate the LOD thresholds of 90% and 95% obtained after the permutation tests. [file Image_3.jpeg]

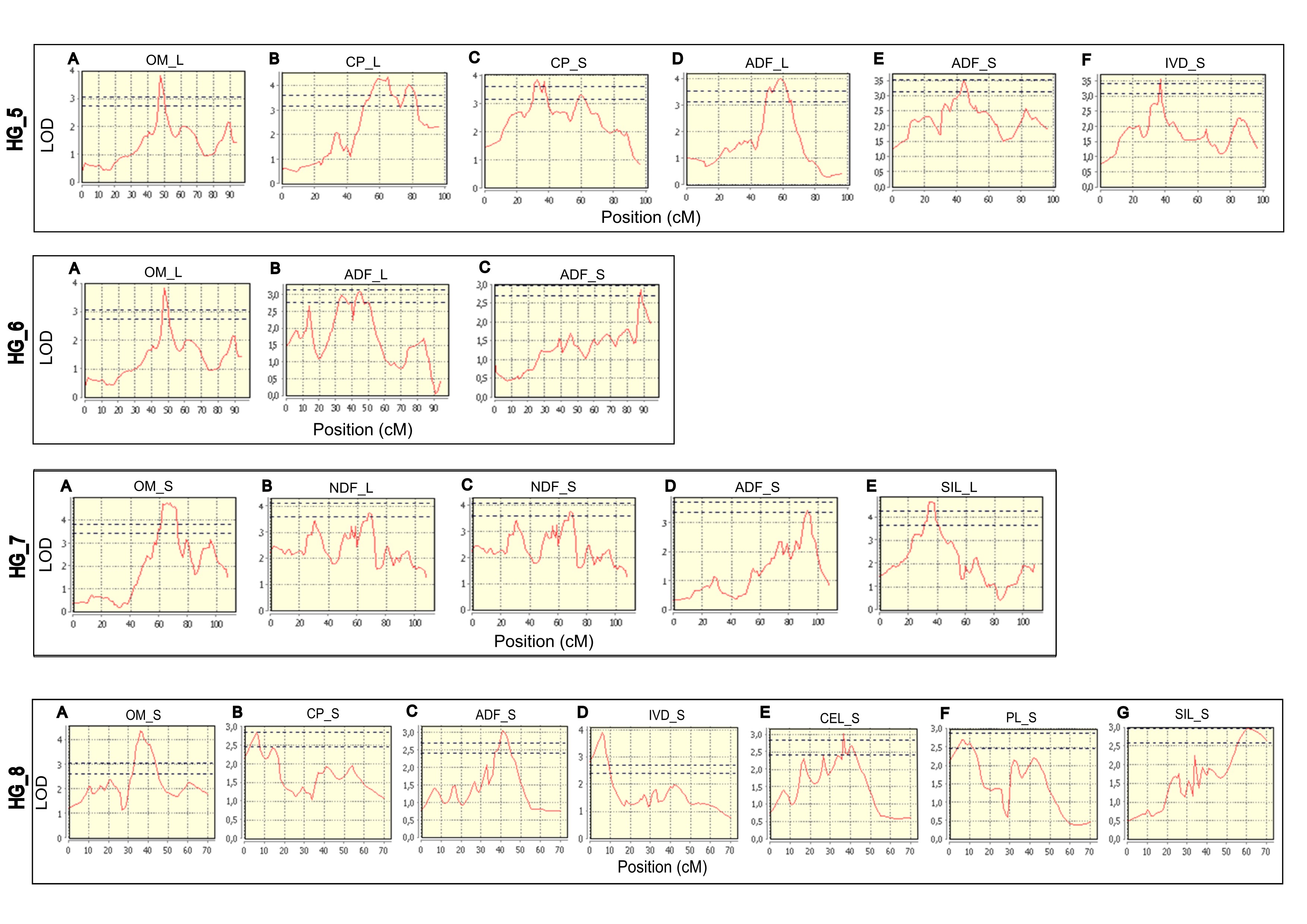

Supplement: Figure S4 — Interval mapping (IM) for forage quality from the guinea grass (Megathyrsus maximus) population in HGs V to VIII. Nutritional quality traits for the leaf (L) and/or stem (S): organic matter (OM_L and OM_S), crude (CP_L and CP_S), in vitro digestibility of organic matter (IVD_S), neutral detergent fiber (NDF_L and NDF_S), acid detergent fiber (ADF_L and ADF_S), cellulose (CEL_S), silica (SIL_L and SIL_S), and permanganate lignin (PL_S). Dotted lines indicate the LOD thresholds of 90% and 95% obtained after the permutation tests. [file Image_4.jpeg]
