# Supplementary material for: High-Resolution Linkage Map With Allele Dosage Allows the Identification of Regions Governing Complex Traits and Apospory in Guinea Grass (Megathyrsus maximus)
Source: Front Plant Sci. 2020 Feb 26;11:15. doi: 10.3389/fpls.2020.00015 (PMC7054243; doi:10.3389/fpls.2020.00015)
Supplement: Supplementary file 5 [file Table_1.docx]

**Table S1.** Reproductive mode of the hybrids from mapping population of guinea grass (*Megathyrsus maximus*).

| Genotype | RM^a^ |  | Genotype | RM |  | Genotype | RM |  | Genotype | RM |
| --- | --- | --- | --- | --- | --- | --- | --- | --- | --- | --- |
| B01 | Apo^b^ |  | **B46** | Apo |  | **B102** | Sex |  | **C24** | Apo |
| B02 | Sex^c^ |  | **B47** | Apo |  | **B103** | Sex |  | **C25** | Apo |
| B03 | ND^d^ |  | **B48** | Apo |  | **B105** | ND |  | **C26** | Apo |
| B04 | ND |  | **B49** | Sex |  | **B108** | Apo |  | **C27** | Sex |
| B06 | Apo |  | **B50** | Sex |  | **B109** | Apo |  | **C28** | Sex |
| B07 | ND |  | **B51** | Apo |  | **B110** | Sex |  | **C30** | Sex |
| B10 | ND |  | **B66** | Apo |  | **B112** | ND |  | **C31** | ND |
| B11 | Apo |  | **B67** | Sex |  | **B113** | Sex |  | **C33** | Apo |
| B12 | Sex |  | **B68** | Apo |  | **B114** | Apo |  | **C34** | Apo |
| B13 | Sex |  | **B70** | Apo |  | **B115** | Apo |  | **C36** | Apo |
| B15 | ND |  | **B71** | ND |  | **B117** | Sex |  | **C37** | ND |
| B16 | ND |  | **B72** | Sex |  | **B118** | Sex |  | **C40** | Apo |
| B18 | Apo |  | **B73** | Apo |  | **B119** | ND |  | **C41** | Apo |
| B20 | Sex |  | **B74** | Sex |  | **B120** | Apo |  | **C43** | ND |
| B21 | Sex |  | **B75** | Sex |  | **B121** | ND |  | **C44** | Apo |
| B22 | Sex |  | **B76** | Apo |  | **B124** | Apo |  | **C45** | Sex |
| B23 | Sex |  | **B77** | ND |  | **B126** | Apo |  | **C47** | Sex |
| B24 | Apo |  | **B80** | ND |  | **B128** | Apo |  | **C48** | Sex |
| B25 | Sex |  | **B81** | Sex |  | **C02** | Apo |  | **C50** | ND |
| B27 | ND |  | **B83** | ND |  | **C03** | Apo |  | **C52** | Apo |
| B28 | Apo |  | **B84** | Sex |  | **C04** | Sex |  | **C53** | Apo |
| B31 | ND |  | **B85** | Sex |  | **C07** | Apo |  | **C54** | Sex |
| B33 | Sex |  | **B91** | Apo |  | **C09** | Apo |  | **C55** | Apo |
| B34 | Apo |  | **B92** | Apo |  | **C10** | Apo |  | **C57** | Apo |
| B35 | Sex |  | **B93** | Apo |  | **C12** | Apo |  | **C58** | Apo |
| B37 | Apo |  | **B94** | Apo |  | **C13** | Sex |  | **C59** | ND |
| B38 | Apo |  | **B95** | ND |  | **C14** | Apo |  | **C60** | Apo |
| B39 | ND |  | **B96** | Sex |  | **C15** | Apo |  | **C62** | Sex |
| B40 | Apo |  | **B97** | Apo |  | **C16** | Sex |  | **C63** | Apo |
| B41 | Apo |  | **B98** | ND |  | **C17** | Sex |  | **C64** | Apo |
| B42 | ND |  | **B99** | Apo |  | **C21** | Apo |  | **C65** | Sex |
| B44 | Apo |  | **B100** | Apo |  | **C22** | Sex |  | **C66** | Sex |
| B45 | Apo |  | **B101** | Apo |  | **C23** | Apo |  | **C90** | ND |

^a^Reproductive mode; ^b^Apomictic; ^c^Sexual; ^d^Not determined.
