# Supplementary material for: High-Resolution Linkage Map With Allele Dosage Allows the Identification of Regions Governing Complex Traits and Apospory in Guinea Grass (Megathyrsus maximus)
Source: Front Plant Sci. 2020 Feb 26;11:15. doi: 10.3389/fpls.2020.00015 (PMC7054243; doi:10.3389/fpls.2020.00015)
Supplement: Supplementary file 6 [file Table_2.docx]

| GM | | | | | | TDM | | | | | | LDM | | | | | |
| --- | --- | --- | --- | --- | --- | --- | --- | --- | --- | --- | --- | --- | --- | --- | --- | --- | --- |
| *G_L_* matrix | | | ***R_L_*** matrix | | | ***G_L_*** matrix | | | ***R_L_*** matrix | | | ***G_L_*** matrix | | | ***R_L_*** matrix | | |
| VCOV | AIC | SIC | VCOV | AIC | SIC | VCOV | AIC | SIC | VCOV | AIC | SIC | VCOV | AIC | SIC | VCOV | AIC | SIC |
| ID | 1610.4 | 1632.8 | ID | 581.7 | 598.5 | ID | 1730.0 | 1752.5 | ID | 864.3 | 886.7 | ID | 1738.9 | 1761.3 | ID | 823.5 | 840.3 |
| DIAG | 1619.6 | 1670.2 | DIAG | 569.4 | 614.4 | DIAG | 1738.2 | 1788.7 | DIAG | 836.7 | 887.2 | DIAG | 1747.5 | 1798.0 | DIAG | 798.6 | 843.5 |
| CS | 581.7 | **598.5** | CS | -272.8 | -239.1 | CS | 871.8 | 888.7 | CS | 216.8 | 250.5 | **CS** | 823.5 | **840.3** | CS | 87.4 | 115.5 |
| CSHet | 587.4 | 632.4 | CSHet | -295.7 | -233.9 | CSHet | 871.0 | 915.8 | CSHet | 170.4 | 232.1 | CSHet | 828.6 | 879.1 | CSHet | 46.4 | 108.1 |
| AR1 | NC | NC | AR1 | -546.0 | -512.3 | **AR1** | **864.3** | **886.7** | AR1 | 241.7 | 275.3 | AR1 | **818.8** | 846.9 | AR1 | NC | NC |
| AR1Het | NC | NC | AR1Het | -568.0 | -506.2 | AR1Het | NC | NC | AR1Het | 168.9 | 230.6 | AR1Het | NC | NC | **AR1Het** | **-92.1** | **-30.4** |
| FA1 | 585.3 | 635.8 | FA1 | NC | NC | FA1 | 870.0 | 926.1 | FA1 | NC | NC | FA1 | NC | NC | FA1 | NC | NC |
| US | **567.3** | 702.2 | **US** | **-713.8** | **-573.3** | US | 873.9 | 1003.0 | **US** | **-9.5** | **130.8** | US | NC | NC | US | NC | NC |
| SDM | | | | | | **PLB** | | | | | | **RC** | | |  |  |  |
| *G_L_* matrix | | | ***R_L_*** matrix | | | ***G_L_*** matrix | | | ***R_L_*** matrix | | | ***G_L_*** matrix | | |  |  |  |
| VCOV | AIC | SIC | VCOV | AIC | SIC |  | AIC | SIC |  | AIC | SIC |  | AIC | SIC |  |  |  |
| ID | 965.4 | 980.1 | ID | 835.8 | 850.5 | ID | 1954.4 | 1971.3 | ID | 1831.0 | **1861.4** | ID | 377.2 | 421.6 |  |  |  |
| DIAG | 966.4 | 990.9 | DIAG | 824.8 | 854.3 | DIAG | 1950.1 | 1995.0 | **DIAG** | **1821.8** | 1872.3 | DIAG | 378.1 | 427.5 |  |  |  |
| CS | 835.8 | **850.5** | CS | 806.6 | **831.2** | CS | 1848.6 | 1871.0 | CS | 1839.5 | 1867.6 | **CS** | **343.0** | **387.3** |  |  |  |
| CSHet | **834.3** | 858.8 | CSHet | 798.0 | 837.4 | CSHet | 1836.1 | 1886.6 | CSHet | 1822.4 | 1878.5 | CSHet | 345.7 | 400.0 |  |  |  |
| AR1 | 836.0 | 850.8 | AR1 | 826.0 | 845.7 | **AR1** | 1839.0 | **1861.4** | AR1 | 1840.8 | 1868.8 | AR1 | 344.9 | 394.2 |  |  |  |
| AR1Het | 834.4 | 859.0 | AR1Het | 815.4 | 849.8 | AR1Het | **1827.5** | 1878.0 | AR1Het | 1823.7 | 1879.9 | AR1Het | 345.7 | 400.0 |  |  |  |
| FA1 | 836.1 | 865.7 | FA1 | NC | NC | FA1 | 1838.1 | 1911.1 | FA1 | NC | NC | FA1 | 346.8 | 406.0 |  |  |  |
| US | 840.3 | 879.6 | **US** | **793.3** | 837.6 | US | NC | NC | US | 1833.3 | 1968.0 | US | 352.1 | 421.1 |  |  |  |

**Table S2.** AIC and SIC values for ***G_L_*** and ***R_L_*** matrices for agronomic traits*****. The lowest values and selected variance-covariance (VCOV) structures are indicated in bold. NC means not converged.

*****Agronomic traits: green matter (GM), total dry matter (TDM), leaf dry matter (LDM), stem dry matter (SDM), percentage of leaf blade (PLB) and regrowth capacity (RC).
