# Supplementary material for: High-Resolution Linkage Map With Allele Dosage Allows the Identification of Regions Governing Complex Traits and Apospory in Guinea Grass (Megathyrsus maximus)
Source: Front Plant Sci. 2020 Feb 26;11:15. doi: 10.3389/fpls.2020.00015 (PMC7054243; doi:10.3389/fpls.2020.00015)
Supplement: Supplementary file 7 [file Table_3.docx]

**Table S3.** AIC and SIC values for ***G_L_*** and ***R_L_*** matrices for nutritional traits*****.

| Leaf | | | | | | | | |
| --- | --- | --- | --- | --- | --- | --- | --- | --- |
|  | **OM_L** | **CP_L** | **NDF_L** | **ADF_L** | **IVD_L** | **CEL_L** | **PL_L** | **SIL_L** |
| AIC | 342.34 | 500.52 | 354.96 | 345.76 | 329.68 | 528.47 | 348.05 | 343.99 |
| SIC | 353.84 | 515.86 | 362.62 | 357.26 | 341.19 | 536.14 | 359.55 | 355.49 |
| Stem | | | | | | | | |
|  | **OM_S** | **CP_S** | **NDF_S** | **ADF_S** | **IVD_S** | **CEL_S** | **PL_S** | **SIL_S** |
| AIC | 328.04 | 310.15 | 1019.01 | 666.55 | 332.18 | 467.56 | 319.97 | 337.44 |
| SIC | 339.44 | 321.55 | 1026.67 | 677.95 | 343.58 | 475.16 | 331.37 | 348.83 |

*****Nutritional quality traits for the leaf and stem: organic matter (OM_L and OM_S), crude protein (CP_L and CP_S), neutral detergent fiber (NDF_L and NDF_S), acid detergent fiber (ADF_L and ADF_S), *in vitro* digestibility of organic matter (IVD_L and IVD_S), cellulose (CEL_L and CEL_S), permanganate lignin (PL_L and PL_S) and silica (SIL_L and SIL_S).
